# Biochemical and Structural Characterization of SplD Protease from *Staphylococcus aureus*

**DOI:** 10.1371/journal.pone.0076812

**Published:** 2013-10-09

**Authors:** Michal Zdzalik, Magdalena Kalinska, Magdalena Wysocka, Justyna Stec-Niemczyk, Przemyslaw Cichon, Natalia Stach, Natalia Gruba, Henning R. Stennicke, Abeer Jabaiah, Michal Markiewicz, Sylwia Kedracka-Krok, Benedykt Wladyka, Patrick S. Daugherty, Adam Lesner, Krzysztof Rolka, Adam Dubin, Jan Potempa, Grzegorz Dubin

**Affiliations:** 1 Department of Microbiology, Faculty of Biochemistry, Biophysics and Biotechnology, Jagiellonian University, Krakow, Poland; 2 Faculty of Chemistry, University of Gdansk, Gdansk, Poland; 3 Department of Analytical Biochemistry, Faculty of Biochemistry, Biophysics and Biotechnology, Jagiellonian University, Krakow, Poland; 4 Haemophilia Biology, Novo Nordisk A/S, Maaloev, Denmark; 5 Department of Chemical Engineering, University of California at Santa Barbara, Santa Barbara, California, United States of America; 6 Department of Computational Biophysics and Bioinformatics, Faculty of Biochemistry, Biophysics and Biotechnology, Jagiellonian University, Krakow, Poland; 7 Department of Physical Biochemistry, Faculty of Biochemistry, Biophysics and Biotechnology, Jagiellonian University, Krakow, Poland; 8 Malopolska Centre of Biotechnology, Krakow, Poland; 9 Center of Oral Health and Systemic Disease, School of Dentistry, University of Louisville, Louisville, Kentucky, United States of America; Aligarh Muslim University, India

## Abstract

*Staphylococcus aureus* is a dangerous human pathogen. A number of the proteins secreted by this bacterium are implicated in its virulence, but many of the components of its secretome are poorly characterized. Strains of *S. aureus* can produce up to six homologous extracellular serine proteases grouped in a single *spl* operon. Although the SplA, SplB, and SplC proteases have been thoroughly characterized, the properties of the other three enzymes have not yet been investigated. Here, we describe the biochemical and structural characteristics of the SplD protease. The active enzyme was produced in an *Escherichia coli* recombinant system and purified to homogeneity. P1 substrate specificity was determined using a combinatorial library of synthetic peptide substrates showing exclusive preference for threonine, serine, leucine, isoleucine, alanine, and valine. To further determine the specificity of SplD, we used high-throughput synthetic peptide and cell surface protein display methods. The results not only confirmed SplD preference for a P1 residue, but also provided insight into the specificity of individual primed- and non-primed substrate-binding subsites. The analyses revealed a surprisingly narrow specificity of the protease, which recognized five consecutive residues (P4-P3-P2-P1-P1’) with a consensus motif of R-(Y/W)-(P/L)-(T/L/I/V)↓S. To understand the molecular basis of the strict substrate specificity, we crystallized the enzyme in two different conditions, and refined the structures at resolutions of 1.56 Å and 2.1 Å. Molecular modeling and mutagenesis studies allowed us to define a consensus model of substrate binding, and illustrated the molecular mechanism of protease specificity.

## Introduction

*Staphylococcus aureus* is a highly prevalent commensal bacterium that transiently or persistently colonizes the nares of 30%–70% of the human population without any detectable adverse effects [1]. However, *S. aureus* is also one of the major human pathogens, being responsible for a broad spectrum of diseases [2]. Staphylococcal infections range from common and relatively harmless ailments such as minor skin infections (boils, abscesses, folliculitis, impetigo) and food poisoning, to life-threatening conditions such as toxic epidermal necrolysis, toxic shock syndrome, osteomyelitis, endocarditis, meningitis, pneumonia, and sepsis [3]. The overall incidence of infections caused only by methicillin-resistant *S. aureus* (MRSA) was reported to be 31.8 cases per 100,000 people per year and the associated mortality rate was 6.3 per 100,000 [4]. The alarming increase in antibiotic resistance observed in hospitals and in community settings in recent years has prompted many studies focusing on staphylococcal physiology [5,6].

The environmental success of *S. aureus* depends on the ability to produce redundant virulence factors, particularly secretory proteases. The proteases, as a group, are of great importance to the virulence of the bacterium [7,8]. Staphylococci are able to secrete up to eight different serine proteases, two cysteine proteases, and one metalloprotease. Individual proteases have diverging roles in the infection process, including inactivation of the host’s protease inhibitors and antimicrobial peptides, modulation of kinin and chemokine synthesis, degradation of immunoglobulins and complement cascade proteins, modification of the bacterial surface, interactions with components of the coagulation and fibrinolysis pathways, and other [9,10,11,12,13,14,15]. However, the specific contributions of the staphylococcal proteolytic system and its individual proteases, except for epidermolytic toxins, to the pathogenicity of *S. aureus in vivo* are still far from being fully understood.

Staphylococcal serine proteases encoded in the *spl* operon are the least characterized of all of the secreted proteolytic enzymes. The operon is located on a pathogenicity island, vSaβ and is adjacent to the genes encoding the enterotoxins and leukocidins, the well-characterized virulence factors [16]. Analysis of 167 clinical isolates of *S. aureus* demonstrated that the complete *spl* operon (containing all 6 Spl protease encoding genes, *splA–F*) is present in the genomes of 31% of isolates. Additionally, 53% of isolates contained incomplete operons encoding 1–5 different Spl proteases. The other 36% of strains had no genes encoding Spl proteases [17]. Like other virulence factors, the *spl* operon is transcribed during the early stationary growth phase, and its expression is regulated by the global accessory gene regulator (agr) [18,19]. The first Spl protease (SplC) was identified in 1997 by high-throughput screening of proteins that cross-reacted with serum from a patient with endocarditis [20]. To date, SplA, SplB, and SplC are the best-characterized Spl proteases in terms of their biochemical and structural properties [21,22,23]. These three enzymes show significant structural homology to V8 protease and epidermolytic toxins, the important virulence factors of *S. aureus*. Except for SplC, for which proteolytic activity has not yet been convincingly demonstrated, SplA and SplB exhibit very limited substrate specificity. So far, the properties of SplD, SplE, and SplF have not been published.

In the present study, we describe the production of active recombinant SplD protease, and its enzymatic and structural properties. We demonstrate its strict substrate specificity using complementary, high-throughput screening methods. The molecular basis of its strict substrate preference is explained using X-ray crystallography, molecular modeling and mutagenesis studies. Finally, we discuss the possible physiological role of SplD in the context of future investigations.

## Materials and Methods

### Expression and purification of SplD protease, its mutants and the protein substrate

A fragment of the *splD* gene encoding the mature protease without the secretion signal peptide was amplified by PCR from genomic DNA of *S. aureus* strain 8325-4, and was cloned into pGEX-5T [24] vector. Ser156Ala, Tyr172Ala and Pro177Gly mutants were obtained by site directed mutagenesis using the template of thrombin cleavable construct. The proteins were expressed in *E. coli* strain BL21(DE3)pLysS (Invitrogen) in Luria-Bertani (LB) broth containing chloramphenicol (34 μg/ml) and ampicillin (100 μg/ml). The cells were cultured at 37 °C until the optical density at 600 nm (OD_600_) reached 0.8 and then at room temperature until the OD_600_ reached 1.2. Protein expression was induced by 1 mM isopropyl β-D-1-thiogalactopyranosid, and culture maintained for another 4 h at room temperature. Bacteria were collected by centrifugation, resuspended in phosphate-buffered saline, and sonicated. The glutathione S-transferase (GST)-SplD fusion protein was purified by affinity chromatography on Glutathione Sepharose (Amersham Biosciences). Four different fusion proteins with linkers engineered to be specifically cleaved by thrombin, CleanCut (Sigma), factor Xa, and SplD were evaluated. Cleavage with thrombin (BioCentrum) was performed on the affinity column in 50 mM Tris-HCl, pH 8.0. The other fusion proteins were eluted from the affinity column and then treated with the relevant protease. All preparations were then dialyzed against 50 mM sodium acetate, pH 5.0. SplD was recovered by ion exchange chromatography on SourceS resin (Amersham Biosciences) followed by gel filtration on Superdex s75pg (Amersham Biosciences) equilibrated with 5 mM Tris-HCl, 50 mM NaCl, pH 8.0 for crystallization, or with 50 mM Tris-HCl, pH 8.0, for long-term storage at -20 °C. All SplD preparations used in this study were of >95% purity, as determined by sodium dodecyl sulfate (SDS)-polyacrylamide gel electrophoresis (PAGE).

A fusion protein containing GST and catabolite repressor protein (CRP) was constructed by cloning the *crp* gene into the pGEX-5T vector. The thrombin cleavage site (PRGS) was replaced with a sequence recognized by SplD (PRWLL↓TSLG; underlined) by site-directed mutagenesis. Protein expression was carried out as described for the GST-SplD fusion protein. The protein was purified to homogeneity by single-step affinity chromatography on Glutathione Sepharose (Amersham Biosciences). GST-RWLLTS-CRP was incubated with SplD in 50 mM Tris-HCl pH 8.0 at a molar ratio of 125:1 for 6 h at 37 °C to achieve >95% hydrolysis. The cleavage site was determined by N-terminal Edman degradation sequencing of the reaction products. 

### Activity assays and initial substrate specificity assessment

The proteolytic activity of SplD and its mutants was detected by zymography [25] using β-casein as a substrate. In brief, the samples were mixed with Laemmli sample buffer without a reducing agent, incubated at room temperature for 15 min, and then separated on a 12% (w/v) polyacrylamide gel containing 0.1% β-casein. After PAGE, the SDS was removed by incubating the gels for 30 min in 2.5% (v/v) Triton X-100. The gels were developed overnight in 100 mM Tris-HCl, pH 7.5 at 37 °C, and stained with 0.1% (v/v) amido-black in 10% (v/v) acetic acid. The zones of hydrolysis were visualized by de-staining the gels in 1% (v/v) acetic acid. The optimum pH and temperature of SplD were determined using soluble β-casein. The extent of cleavage of the reporter protein was monitored by SDS-PAGE.

Hydrolysis of native proteins was evaluated by incubation with SplD at a 1:1 molar ratio for 24 h in 100 mM Tris-HCl, pH 8.0, at 37 °C and the reaction products were analyzed by SDS-PAGE. The following proteins were tested: chicken egg white lysozyme, ovalbumin, soyabean trypsin inhibitor, goat immunoglobulins (IgGs), bovine serum albumin (BSA), GST, β-casein, cytochrome c, carbonic anhydrase, human lactoferrin, human serum transferrin, and human fibrinogen. Hydrolysis of synthetic chromogenic and fluorogenic substrates (Table S1 in File S1) was evaluated in the same conditions as for the native proteins.

### Edman degradation and matrix-assisted laser desorption/ionization–time of flight (MALDI–TOF) mass spectrometry (MS)

The products of hydrolysis of native protein substrates by SplD were analyzed by N-terminal Edman degradation sequencing (BioCentrum) of peptides separated by SDS-PAGE and immobilized on polyvinylidene fluoride membranes, or by MALDI-TOF MS.

### Positional scanning synthetic combinatorial library (PS-SCL)

The P1 substrate preference of SplD was determined using a combinatorial library of synthetic, aminomethylcoumarin (AMC)-labeled peptide substrates, as previously described [26]. In brief, 16 sub-libraries were constructed with a general structure of Ac-P4-P3-P2-P1-AMC; each sub-library contained a fixed natural amino acid (except Met, Cys, Gly, or Trp) at the P1 position and an equimolar mixture of those amino acids at further positions. All sub-libraries were tested at a substrate concentration of 9 mM in 50 mM Tris-HCl, pH 8.0. The active enzyme concentration was 1 μM. Enzymatic activity was monitored as an increase in fluorescence emission at 455 nm (380 nm excitation).

### Libraries of synthetic tetrapeptide substrates (LSTS)

The LSTS are composed of fluorescence-quenched substrates of a general structure ABZ-X4-X3-X2-X1-ANB-NH_2_ where 5-amino-2-nitrobenzoic acid (ANB) quenches the fluorescence of aminobenzoic acid (ABZ). The library preparation and one of the possible modes of selection using the release of quenched fluorescence following peptidyl moiety hydrolysis by the test protease were performed as previously described [27,28,29]. In brief, each of 19 X4 fixed sub-libraries (1 mg/ml; positions X3-X1 contained equimolar mixtures of tested residues) was incubated with the enzyme (14.4 μM), and the increase in fluorescence was monitored (excitation, 320 nm; emission, 410 nm). X4 was fixed, being the residue of the most active sub-library, and 19 X3 fixed sub-libraries were constructed; the selection was repeated to determine the most preferred X3 residue. Further positions were deconvoluted in a similar manner.

The second mode of selection involved monitoring of ANB-NH_2_ release by recording the increase in absorbance. The major difference between the two modes of selection is based on the fact that, in the second mode, the X4–X1 positions of the initial library correspond to positions P4–P1 of the substrate, but in the first mode, each iteration may select substrates hydrolyzed at several different sites (Note S1 in File S1). ANB release-based selection was performed as follows: 19 sub-libraries were prepared, each containing 1 of the 19 natural amino acid residues (except Cys) at position P4, while positions P3–P1 contained equimolar mixtures of these residues. Each sub-library (3 mg/ml final concentration) was incubated for 90 min with the enzyme (1.15 μM) and the increase in absorbance was monitored at 410 nm. P4 was then fixed with the residue corresponding to the most efficiently hydrolyzed substrate. Then, 19 sub-libraries were synthesized, in each of which the P3 position contained 1 of the 19 natural amino acid residues and positions P2 and P1 contained equimolar mixtures of these residues. The procedure was iterated until all of the positions of the most efficiently hydrolyzed substrate were determined. 

The substrates that were most efficiently hydrolyzed in both selection processes were resynthesized. The hydrolysis sites were determined by high-performance liquid chromatography (HPLC)-MS and the kinetics of hydrolysis were measured as previously described [30,31].

### Cellular library of peptide substrates (CLiPS)

The complete consensus sequence that was recognized and cleaved by SplD was determined using a CLiPS methodology, as previously described [32]. In brief, a library of 6 × 10^7^ *E. coli* clones, each displaying a surface bait composed of streptavidin peptide ligand and a substrate sequence consisting of eight consecutive, randomized amino acids and the SH3 domain binding peptide, was screened for proper bait display and SplD hydrolysis using fluorescence-activated cell sorting. First, the clones exhibiting both red and green fluorescence after incubation with phycoerythrin-conjugated streptavidin (50 nM) and SH3 domain-conjugated green fluorescence protein (250 nM) were selected. The selected clones were incubated for 2.5 h with 5.5 μM SplD in 0.2 M Tris-HCl, pH 7.6. Clones expressing substrates that were specifically hydrolyzed were isolated by sorting red cells only. After several consecutive rounds of sorting while gradually decreasing the incubation time from 2.5 h to 40 min, the cleavage of individual clones was analyzed by incubation with 5.5 μM SplD for 30 min. The hydrolyzed clones were sequenced (Table 1; Table S2 in File S1) and the data were analyzed to determine the consensus sequence recognized by SplD.

**Table 1:** Experimentally determined SplD cleavage sites within proteins and peptides.

	P4	P3	P2	P1	P1'
Bovine β-casein	L	A	**L**	A	R
	K	**Y**	**P**	**V**	E
	L	T	**L**	**T**	D
	P	F	A	Q	T
	P	V	V	**V**	P
GST	A	**W**	**P**	**L**	Q
GST-WELQ-SplD	P	**W**	E	**L**	Q
Synthetic substrate	A	A	**P**	**L**	pNA
CLiPS	T	**Y**	**P**	**I**	**S**
	V	**Y**	G	**I**	**S**
	L	**Y**	**P**	**I**	**S**
	**R**	**W**	**L**	**L**	**S**
	T	H	I	**L**	**S**
	**R**	**Y**	**L**	**L**	**T**
	Q	**W**	**L**	**L**	A
	Q	**Y**	**L**	**L**	G
	V	**Y**	S	**T**	**S**
	**R**	**Y**	W	**T**	**S**
	**R**	**Y**	**P**	**T**	**S**
	**R**	**W**	**P**	**T**	**S**
	**R**	**Y**	**L**	**T**	**S**
	**R**	**Y**	**L**	**T**	G
	**R**	**Y**	**P**	**T**	**S**
	L	F	**P**	**V**	**S**
	V	**Y**	**P**	**V**	D
	M	**W**	Q	**V**	**S**
Consensus sequence	**R**	**Y/W**	**P/L**	**T/L/I/V**	**S**
Fusion protein (RWLLTS)	**R**	**W**	**L**	**L**	**T**

Residues corresponding to the consensus sequence are highlighted bold. Cleavage products of β-casein, GST, GST-Q-SplD and RWLLTS fusion protein were identified by mass spectrometry and Edman degradation sequencing. Prediction of cleavage sites within CLiPS determined sequences is based on sequence alignment, PS-SCL and LSTS data and experimental analysis of cleavage of a consensus sequence engineered into a fusion protein. In case of CLIPS substrates only those corresponding to the consensus are depicted – for a full list of substrates selected using CLIPS see Table S2 in File S1.

### Crystallization and structure determination

SplD was concentrated to 40 mg/ml by ultrafiltration in 5 mM Tris-HCl, 50 mM NaCl, pH 8.0. Sitting drop vapor diffusion screening was performed at room temperature (~20 °C). After several weeks, crystals appeared in the Index 39 (0.1 M HEPES pH 7.0, 30% Jeffamine ED-2001) and Index 95 (0.1 M KSCN, 30% Polyethylene glycol monomethyl ether 2,000) solutions (Hampton Research). Further optimization yielded the best crystals in 30%–32.5% Jeffamine ED-2001 in 0.1 M HEPES, pH 6.5–7.5 and 30%–33% PEG 2000 monomethyl ether containing 0.1–0.5 M KSCN. SplD crystals were cryopreserved in 25% (v/v) glycerol in mother liquor and flash-cooled in liquid nitrogen. The diffraction data were collected at 100 °K on a Rigaku MicroMax 007HF rotating anode diffractometer. Data were indexed and integrated using MOSFLM software [33]. Further computational steps were performed using programs contained in the CCP4 software package [34] . Data were scaled using SCALA [35,36]. Molecular replacement was performed using Phaser software [37] with an alanine search model based on the structure of SplA (Protein Data Bank [PDB] code 2W7S). The structures were refined in multiple rounds of manual model building and restrained refinement, which were performed using COOT [38] and Refmac 5.0 [39] software, respectively. Throughout the refinement, 5% of the reflections were used for cross-validation analysis [40], and the behavior of R_free_ was used to monitor the refinement strategy. In the final steps of refinement, water molecules were added using Arp/Warp [41] and were manually inspected. The final models were deposited in PDB under accession numbers 4INK and 4INL. Data collection and refinement statistics are summarized in Table 2.

**Table 2:** Data collection and refinement statistics.

PDB ID	**4INK**	**4INL**
**Data Collection**		
Space group	F432	F432
Cell constants (a=b=c) (Å)	175.56	174.27
Wavelength (Å)	1.542	1.542
B factor (Wilson) (Å^2^)	20.7	29.6
Resolution range (Å)	27.76 - 1.56	26.27- 2.10
Completeness (%)	98.2 (94.9)	86.7 (72.1)
R_merge_ (%)	0.053 (0.255)	0.076 (0.159)
R_meas_ (%)	0.057 (0.278)	0.089 (0.205)
Observed reflections	233543 (28416)	35580 (2464)
Unique reflections	32697 (4508)	11885 (1394)
I/σ(I)	15.6 (4.1)	11.0 (3.1)
Average multiplicity	7.1 (6.3)	3.0 (1.8)
**Refinement**		
Resolution (Å)	25.0 - 1.56	20.0 - 2.10
Number of reflections used	30930	11303
R-factor (%)	17.9	21.2
R_free_ (%)	21.0	25.6
Average B (Å^2^)	20.38	31.36
**RMS from ideal values**		
Bond length (Å)	0.012	0.011
Bond angles (°)	1.467	1.529
**Ramachandran statistics (%)**		
Most favored regions	96.0	96.0
Additionally allowed regions	4.0	4.0
Generously allowed regions	0	0
**Content of asymmetric unit**		
Number of protein molecules/residues/atoms	1/203/1595	1/201/1510
Number of solvent molecules	329	66

Statistics for the highest shell are listed in parentheses.

### Modeling of substrate binding

Computer model of a short peptide (H-WLTS-OH) spanning the consensus sequence recognized by SplD, was constructed and optimized in the MMFF94 force field using Avogadro software [42,43]. Substrate peptide was manually placed at the active site cleft of SplD by analogy to canonical inhibitors [44,45,46]. The resulting system was hydrated (~12,000 water molecules) and charge neutralized. The system was minimized, protein and substrate positions were constrained, and two 100 ps equilibrating molecular dynamics (MD) simulations were performed, with the first in the NVT ensemble, and the second in the NpT ensemble. Constraints on the protein and the substrate were removed and the molecular dynamics of the system was simulated over 5 ns. CHARMM 2.7 parameters were used for the protein, substrate, and ions [47,48], while transferable intermolecular potential 3 point (TIP3P) parameters [49] were used for water. Long-range electrostatic interactions were accounted for using the Particle Mesh Ewald (PME) summation method [50]. Simulations were carried out at a constant pressure (1 atm) controlled using the Parrinello–Rahman method [51]. The temperature (310 K) was controlled independently using a modified Berendsen thermostat [52]. Periodic boundary conditions with minimum image convention and a cutoff of 10 Å were used in all three directions. The time step was set at 2 fs. The simulations and data analyses were performed using GROMACS 4.5.3 software [53,54]. Hydrogen bonds were defined according to the following criteria: a distance between a hydrogen bond donor (d) and an acceptor (a) of ≤3.5 Å, and an angle between the a–d vector and the d–H bond of ≤30 °.

### Bioinformatic analysis

Human (*Homo sapiens*) and staphylococcal (*S. aureus*) proteomes were analyzed for the presence of consensus sequences recognized by SplD using BLAST searches of the Uniprot database. Initial hits were manually verified in the context of known niches of *S. aureus* commensal colonization and pathological infection. The steric accessibility of the potential cleavage sites was taken into account if information was available. 

## Results

### Production of recombinant SplD protease

The recombinant SplD protease was obtained by heterologous expression in *E. coli* as a GST fusion protein. Approximately 1 mg of purified protein was obtained per 1 L of starting culture. The initial constructs were engineered to remove the purification tag by using factor Xa or CleanCut protease to generate recombinant SplD with the native N-terminus (NH_2_-Glu(1)-Asn-Ser(3)-...). To achieve this, the thrombin cleavage site in the pGEX-5T vector (LVPR↓GS-E(1)) was replaced with a factor Xa site (IEGR↓-E(1)) or a CleanCut site (LVPWELQ↓-E(1)) by site-directed mutagenesis. The fusion protein with the factor Xa cleavage site was not processed by factor Xa, even after prolonged incubation. By contrast, the latter fusion protein was efficiently cleaved in the presence of CleanCut protease, but N-terminal sequence analysis of the final product revealed glutamine instead of the expected glutamic acid at the N-terminus (Q-SplD). This cleavage at the CleanCut site (LVPWEL↓Q-E(1)) was also observed when incubated without the external protease, which indicates autoproteolysis of the fusion protein by SplD itself. Therefore, to exploit this autoprocessing to generate recombinant SplD with a native N-terminus, the cleavage site was modified to a LVPWEL-E(1) sequence. Unfortunately, this fusion protein underwent slow autoproteolysis within the GST moiety (AWPL↓Q), some distance from the expected cleavage site. 

Previous studies of SplB protease confirmed the importance of appropriate N-terminal processing for enzymatic activity [21]. However, Q-SplD and GS-SplD, which were obtained by autoprocessing or thrombin processing of the fusion proteins, were active in the zymography assay with β-casein as a substrate, and were considered suitable for further characterization (see Discussion). Therefore, all further analyses were performed using GS-SplD (and its mutants), a recombinant SplD protease containing the Gly-Ser dipeptide at the N-terminus, as compared with mature SplD obtained from staphylococci.

### Initial characterization of SplD proteolytic activity

Once the activity of recombinant SplD had been confirmed by zymography, the cleavage of multiple different native proteins and synthetic substrates was analyzed to determine the substrate specificity of the protease. The optimum pH and temperature of SplD were determined using β-casein as the substrate (pH 8.0; 37 °C). Analysis of the effects of SplD on native proteins revealed appreciable activity against β-casein and GST, but not other proteins, despite testing excess enzyme concentrations and prolonged incubation times. The cleavage products were identified by MS and N-terminal sequence analysis. The identified cleavage sites are summarized in Table 1. SplD did not cleave several proteins, including chicken egg white lysozyme and ovalbumin, soybean trypsin inhibitor, goat IgG fraction, BSA, cytochrome c, carbonic anhydrase, human lactoferrin, human serum transferrin, and human fibrinogen.

Hydrolysis of a number of synthetic substrates was also evaluated. Because the S1 family proteases are specific for P1 substrate residues [55], the tested compounds included substrates with P1 residues corresponding to the SplD cleavage sites in GST and β-casein. In addition, we also examined the hydrolysis of other commercially available substrates to extend the repertoire of tested sequences. Of 33 compounds tested (Table S1 in File S1), we only detected the hydrolysis of Suc-Ala-Ala-Pro-Leu-pNa. However, because the reaction was inefficient, kinetic studies and use of the substrate for routine detection of activity were considered unfeasible.

Although multiple substrates were tested under favorable conditions, very few were actually hydrolyzed. Therefore, these initial studies prompted our preliminary conclusion that the substrate specificity of the SplD protease is limited and is probably determined at the S1 and other subsites.

### Determination of SplD substrate preference at nonprimed positions using combinatorial libraries of synthetic peptide substrates

To comprehensively determine the preference of SplD for residues at the P4-P1 substrate positions, the protease was probed with LSTS, with a general structure of ABZ-P4-P3-P2-P1-ANB-NH_2_. The libraries were deconvoluted, starting from P4, by monitoring the absorbance of the released ANB, until SplD preference for all tested substrate positions was determined.

**Figure 1:**
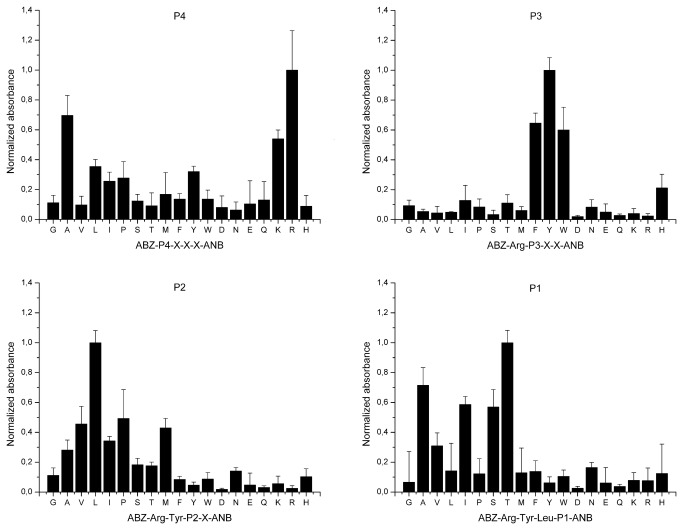
Substrate specificity of SplD at P4-P1 subsites. Substrate preference of SplD protease was determined using libraries of synthetic tetrapeptide substrates (LSTS). Libraries of a general structure ABZ-P4-P3-P2-P1-ANB-NH_2_ were deconvoluted starting form P4 position as described in Materials and Methods. Vertical bars indicate the activity of the enzyme (absorbance of released ANB-NH_2_) against particular sub-libraries normalized to the most active sub-library in each library. Residues fixed at particular subsites (designated at the top of each panel) are indicated with the single-letter amino acid code. X indicates randomized substrate position.

SplD selected amino acids with a small, hydroxyl group containing side chains (threonine and serine) and aliphatic side chains (alanine, isoleucine, and valine) at position P1 (Figure 1), with threonine being the preferred residue. These results confirm and substantiate the data obtained by analyzing the cleavage sites in native proteins, as described above (Table 1). At the other subsites, leucine was preferred at P2, but other aliphatic side chains and proline were also accepted. Unusually for the S1 family of proteases, SplD showed very limited substrate preference at P3, where the aromatic side chains of tyrosine, phenylalanine, and tryptophan were almost exclusively selected. Residues of different physicochemical properties were selected at the P4 subsite, including positively charged (arginine and lysine), aliphatic (alanine, isoleucine, and leucine), and aromatic (tyrosine) residues or proline, but arginine-containing substrates were hydrolyzed most efficiently.

The P1 substrate residue is the primary determinant of specificity for the S1 family of proteases. To evaluate whether the direction of substrate deconvolution or the identity of the reporter group affected the result of the LSTS screening, we reevaluated SplD preference at P1 using a different experimental approach, the PS-SCL. Sixteen sub-libraries with a general structure of acetyl-P4-P3-P2-P1-AMC, with a fixed residue at P1 position and an equimolar mixture of the tested residues at positions P2–P4, were incubated with the protease and hydrolysis of the P1-AMC bond was monitored as an increase in AMC-specific fluorescence.

**Figure 2:**
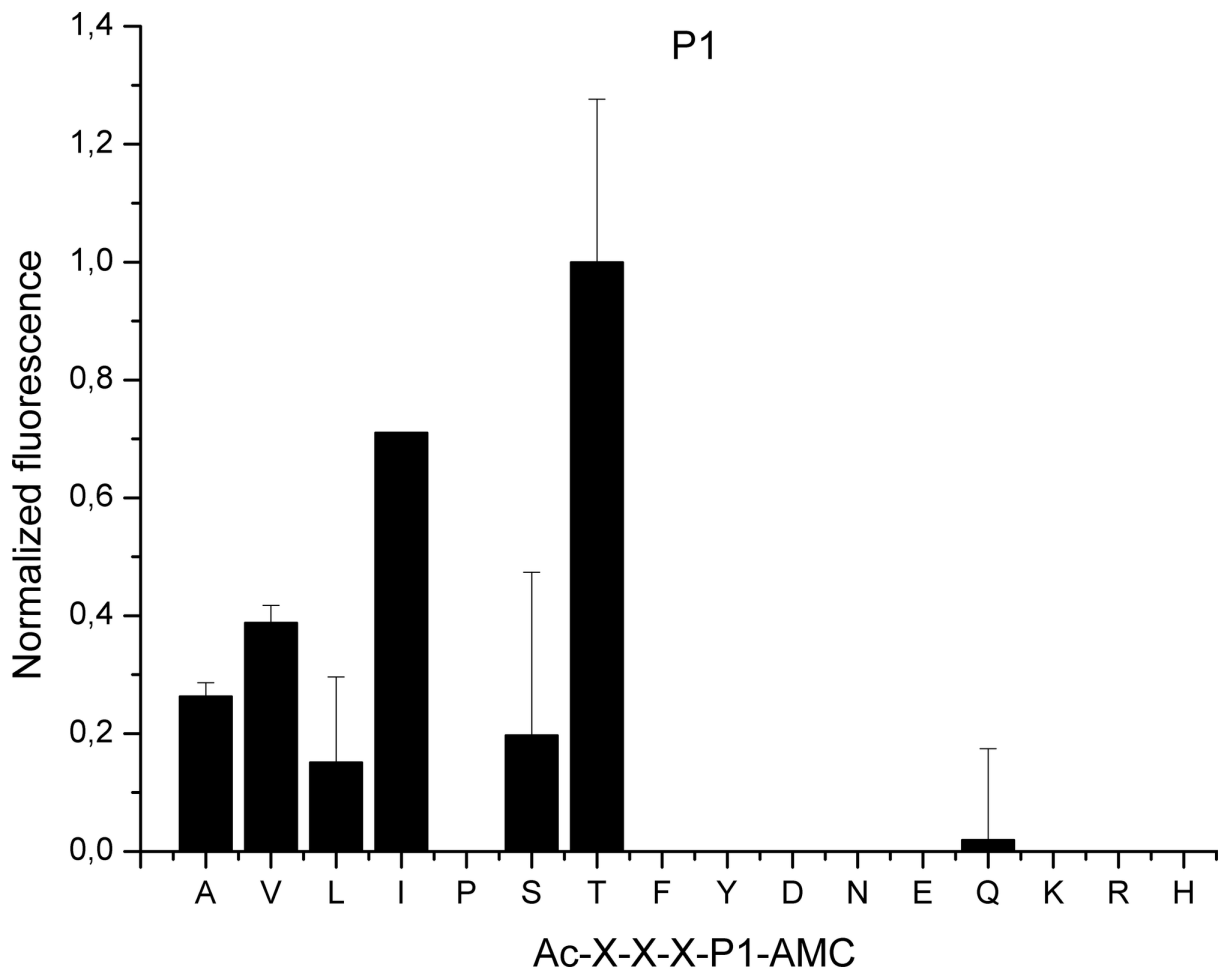
Substrate specificity of the SplD protease at the P1 subsite. Substrate preference of SplD at P1 subsite was determined using a positional scanning synthetic combinatorial library (PS-SCL) of a general structure Ac-P4-P3-P2-P1-AMC as described in Materials and Methods. Vertical bars indicate the activity of the enzyme against each tested sub-library (fluorescence of released AMC) normalized to the most active sub-library. Residues fixed at P1 subsite are indicated with the single-letter amino acid code. X indicates randomized substrate position.

Despite the different direction of deconvolution, the results obtained using the PS-SCL approach substantiated the results obtained using LSTS. SplD tended to hydrolyze substrates accommodating amino acids with a small, hydroxyl group containing (threonine and serine) or aliphatic side chains in the P1 position (Figure 2). Again, threonine was the preferred residue. SplD failed to recognize substrates containing residues with aromatic or charged side chains, or proline. The only exception was glutamine, but the substrates containing this residue at P1 position were inefficiently hydrolyzed.

### Selection of an efficient fluorescence-quenched synthetic substrate of SplD using the LSTS approach

Using libraries of the same general structure (ABZ-X4-X3-X2-X1-ANB-NH_2_) but a different selection approach, we identified a sensitive, synthetic substrate for SplD. The library was deconvoluted starting from the P4 position by monitoring the release of quenched fluorescence.

**Figure 3:**
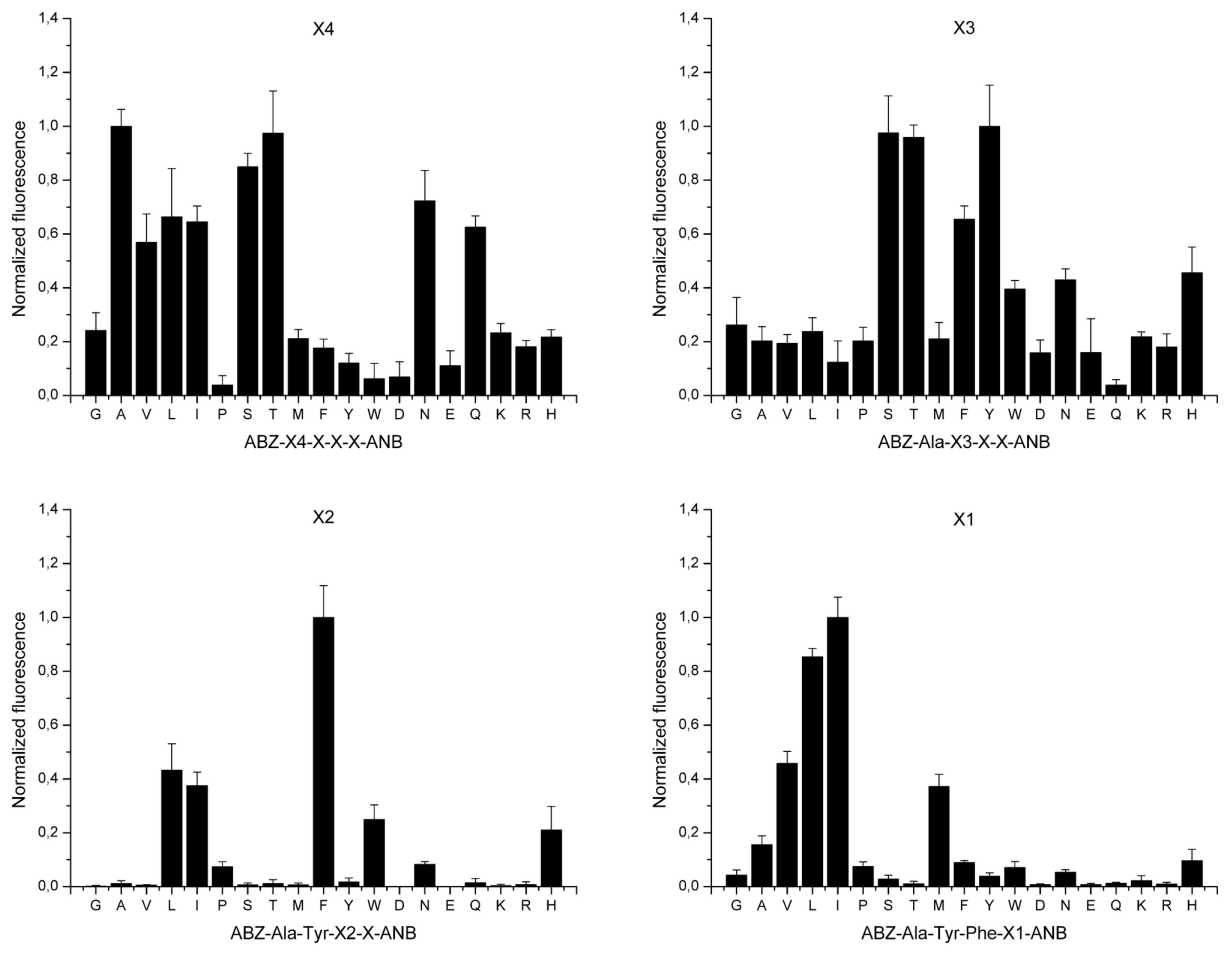
Selection of an efficient fluorescence-quenched substrate of the SplD protease. Synthetic tetrapeptide substrate libraries of a general structure ABZ-X4-X3-X2-X1-ANB-NH_2_ were screened for efficient fluorescence-quenched substrates of SplD as described in Materials and Methods. Vertical bars indicate the activity of the enzyme against a particular sub-library (released fluorescence) normalized to the most active sub-library in each library. Residues fixed at particular subsites (indicated at the top of each panel) are designated with the single-letter amino acid code. X indicates randomized substrate position.

In the X4 fixed library, the sub-libraries containing alanine, threonine, and serine were hydrolyzed most efficiently, while those containing asparagine, glutamine, leucine, isoleucine, and valine were also efficiently cleaved (Figure 3). In the Ala-X3 fixed library, the sub-libraries containing serine, threonine, tyrosine, and phenylalanine residues were preferentially cleaved. In the Ala-Tyr-X^2^ fixed library, the sub-library containing phenylalanine was preferred, but leucine, isoleucine, histidine, and tryptophan were also accepted. Finally, SplD strongly selected isoleucine, leucine, valine, and methionine in the P3’ position (Ala-Tyr-Phe-X1 fixed library). In this selection scheme, the X(n) positions of the library do not correspond to the P(n) positions of the substrate, except for X1, which corresponds to P3’. This is discussed in more detail in Note S1 in File S1.

The optimal substrates determined with the absorbance- and fluorescence-based selection approaches were resynthesized and the kinetics of their hydrolysis by SplD were determined (Table 3). The cleavage sites were verified by HPLC-MS. ABZ-Ala-↓-Tyr-Phe-Ile-ANB-NH_2_ was hydrolyzed more efficiently than ABZ-Arg-Tyr-Leu-Thr-↓-ANB-NH_2_. We believe that the lower rate of hydrolysis of ABZ-Arg-Tyr-Leu-Thr-ANB-NH_2_ is due to the unnatural moiety in the P1’ position rather than the preference of SplD to the peptidyl part of the tested substrates.

**Table 3:** Kinetics of hydrolysis of SplD substrates.

**Substrate**	Library (selection mode)^*^	**K_m_ [µM]**	**k_cat_ [s^-1^]**	**k_cat_/K_m_ [M^-1^ s^-1^]**
ABZ-Ala-↓-Tyr-Phe-Ile-ANB-NH_2_	LSTS (fluorescence)	13.1±0.2	15.2±3.1	1,169,200
ABZ-Arg-Tyr-Leu-Thr-↓-ANB-NH_2_	LSTS (absorbance)	87.3±14.1	3.1±0.9	35,509
ABZ-Trp-Leu-Thr-↓-Ser-ANB-NH2	CLIPS	14.7±2.7	1.5±0.1	102,772
ABZ-Trp-Leu-Val-↓-Ser-ANB-NH2	CLIPS	31.1±5.1	1.9±0.2	59,768

* ^for detailed description see Materials and Methods^

### Comprehensive analysis of SplD substrate specificity using CLiPS

Both PS-SCL and LSTS are composed of short, synthetic peptides with bulky reporter groups. Moreover, both methods rely on deconvolution to determine the protease preference at subsequent positions of a substrate, and poorly reflect the effects of subsite cooperativity on the rate of substrate hydrolysis. To verify the substrate preference of SplD against protein substrates, and to simultaneously account for subsite cooperativity, we used a high-throughput CLiPS substrate display and selection method [32]. The preferentially cleaved substrates were selected from a pool of ~10^8^ random sequences displayed in the context of an *E. coli* surface protein. Testing a large number of permutations within the eight amino acid randomized sequence allowed us to determine the substrate preference of the protease at the P(n) and P(n)’ subsites. The sequences that were most efficiently hydrolyzed by SplD after multiple rounds of CLiPS selection are summarized in Table 1 (Table S2 in File S1). Alignment of the resulting sequences revealed a consensus of five consecutive amino acid residues, R-(Y/W)-(P/L)-(T/L/I/V)-S, which was recognized and efficiently cleaved by SplD.

The CLiPS method does not allow for direct determination of the cleavage site within the consensus sequence. However, comparison of the PS-SCL and LSTS profiles of SplD specificity and the CLiPS consensus sequence clearly indicates that the enzyme should specifically hydrolyze the (T/L/I/V)-S peptide bond. To confirm this possibility, we constructed a substrate composed of two globular partners connected by a linker containing a variant of the consensus sequence determined in CLiPS (RWLLTS). In the light of LSTS profiling results, this variant has an ambiguous cleavage site, either at the T-S peptide bond as suggested by P1 specificity or at L-T peptide bond as suggested by P3 and P4 specificity. It was purposely chosen to determine the influence of bulky and charged residues that are likely to be selected at positions P3 and P4. SDS-PAGE confirmed that SplD cleaved this engineered substrate into two peptides. N-terminal sequence analysis of cleavage products revealed hydrolysis of the Leu–Thr peptide bond within the consensus sequence, confirming SplD specificity at P3 and P4 subsites. Therefore, the data obtained using synthetic peptide substrate libraries was confirmed by analyzing the specificity of SplD using protein substrates. Thus, it is clear that SplD is a highly specific staphylococcal extracellular protease that recognizes and efficiently hydrolyzes substrates containing the consensus sequence motif R-(Y/W)-(P/L)-(T/L/I/V)↓S.

### Crystal structure of SplD

To explain the molecular basis of the limited substrate specificity observed in biochemical assays, we crystallized and solved the structure of SplD. Crystals were obtained under two different crystallization conditions, and diffracted to resolutions of 1.56 Å and 2.10 Å. The data collection and the refinement statistics are summarized in Table 2. Because both structures belong to the same space group and are essentially identical (RMSD of 0.24 Å for all Cα atoms) only the high-resolution structure (PDB ID: 4INK) is discussed in this report. The lower resolution structure (4INL) is provided for reference, and confirms that the structural details are not dependent on the crystallization conditions.

SplD has a chymotrypsin-like fold, typical of the S1 family of proteases. The molecule consists of two β-barrel domains with roughly perpendicular axes [56] (Figure S1 in File S1). The active site is located in the interface between the domains. Domain I is formed of residues Tyr15–Phe99 and the C-terminal part of the protein (Ser190–Arg203). Domain II is primarily composed of C-terminal residues (Pro114–Phe189) and a small N-terminal fragment (Glu1-Ile7). An extended segment encompassing residues Thr100–Glu113 links the two domains. The entire molecule is well-defined by its electron density, except for the side chain of the Glu1 and residues Ser0 and Gly(-1) and the three solvent-exposed side chains of lysine residues.

The high level of amino acid sequence homology with other staphylococcal serine proteases is reflected by the structural similarities. Superimposing the SplD structure with known structures of other *spl* operon proteases yielded a RMSD of 0.93 Å (for 184 equivalent Cα atoms) for SplC, 1.08 Å (192) for SplB, and 1.00 Å (184) for SplA. The structures of epidermolytic toxins A and B, and V8 protease are also closely related, with RMSD values of 1.77 Å (181), 1.91 Å (178), and 1.17 Å (170), respectively. Despite the low primary structure identity, SplD can be superimposed on the type protease of the S1 family (chymotrypsin) with a relatively low RMSD of 2.01 Å (177).

Differences between the discussed structures are primarily manifested in the arrangement of the surface loops, particularly loops A (Trp20–Thr25) and D (Pro121–Gln129), and in a region encompassing Val84–Thr100. Of particular significance are the differences in loops C (Pro74–Asp78) and 3 (an α-turn in SplD), which are determinants of substrate specificity [45,57], and loop 1 (a γ-turn in SplD), a component of the S1 cavity.

### Catalytic machinery

**Figure 4:**
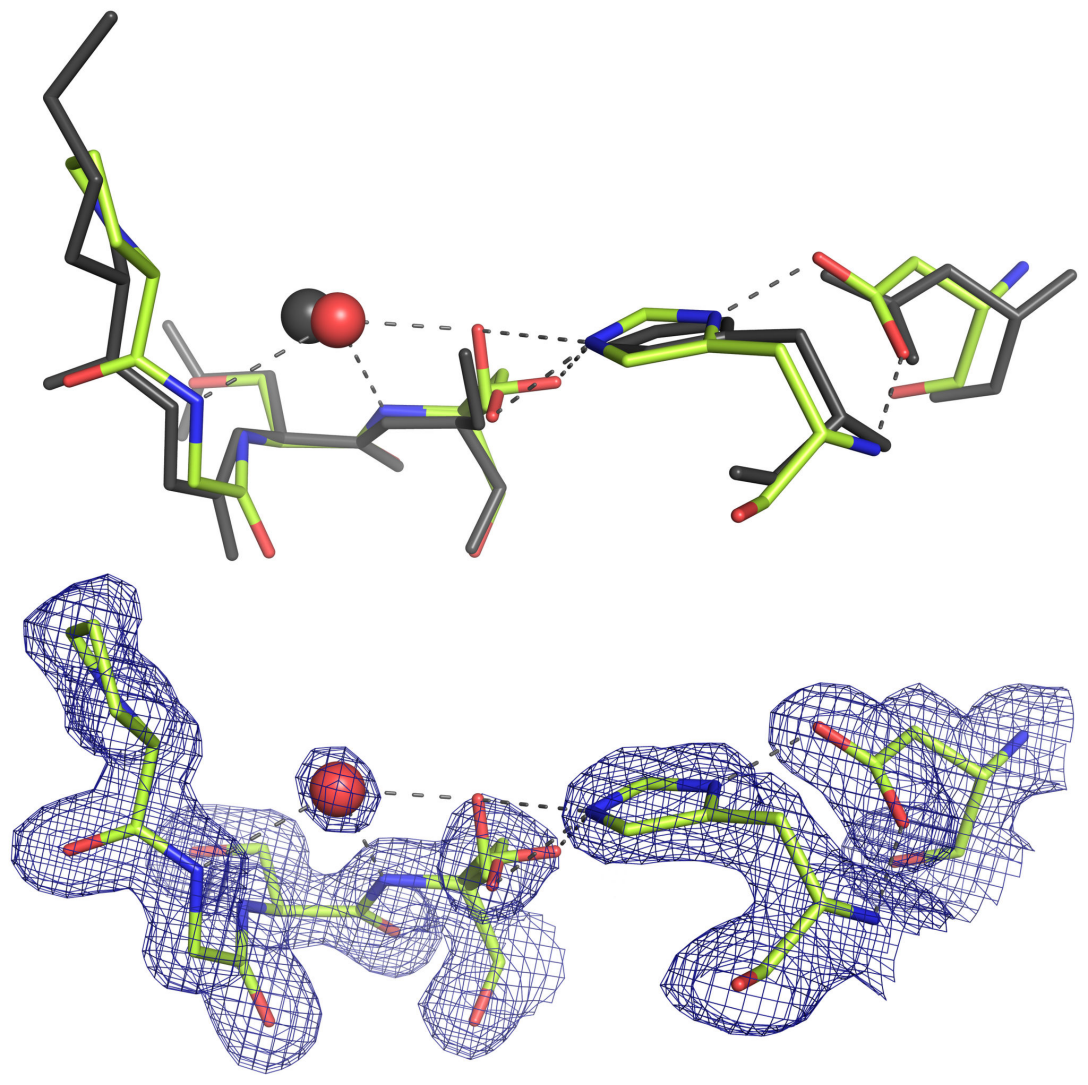
The crystal structure of SplD demonstrates canonical conformation of the catalytic triad and the oxyanion hole. (Upper panel) Catalytic triad residues and the main chain fragment constituting the oxyanion hole of SplD (limon) superposed with corresponding residues of chymotrypsin (black). (Lower panel) Electron density (contoured at 1.1σ) around SplD fragment depicted in the upper panel. Red sphere represents a water molecule. Dashed lines represent hydrogen bonds.

The most prominent features of the catalytic machinery of the S1 family of serine proteases include a characteristic spatial arrangement of side chains of the catalytic triad residues (Asp102, His57, and Ser195; chymotrypsin numbering) which provides the nucleophilic property of serine O^γ^ [46], and an oxyanion hole, a charge-compensating structure formed by the backbone amides of the catalytic triad serine (n) and n-2 residues [58]. Both of these features are present in the structure of SplD. The equivalent residues are clearly defined by their electron densities. In the catalytic triad, the side chain carboxyl oxygen of Asp78 forms a canonical, short (2.53 Å) hydrogen bond with N^δ^ of His39. The side chain of Ser156 is found in three alternative orientations (*gauche+*, *gauche*- and *trans*; Note S2 in File S1; Figure S2 in File S1). In the canonical *gauche*+ orientation, the O^γ^ of the catalytic triad serine forms a hydrogen bond with N^ε^ of the catalytic triad histidine. The catalytic triad residues of SplD can be superimposed on the residues of V8 protease with an RMSD of 0.18Å (Figure 4). Overall, the catalytic triad of SplD assumes a configuration that is typical of the active serine proteases of the S1 family, demonstrating high conservation of this crucial structure.

The structure of SplD reveals canonical arrangement of the oxyanion hole formed by the backbone amide hydrogen atoms within the Pro153–Gly154 and Ser155–Ser156 (catalytic triad serine) peptide bonds. In SplD, Pro153 has main chain angles of *ϕ* = -42 ° and ψ = 135 °, which correspond to those of the equivalent Gly166 residue in V8 protease (PDB ID 1qy6; *ϕ* = -50 ° and ψ = 130 °). Gly154 has main chain angles of *ϕ* = 153 ° and ψ = -32 °, which are similar to those of the corresponding Gly167 in V8 protease (*ϕ* = 150 ° and ψ = -29 °) (Figure 4; Table S3 in File S1). This is important because the oxyanion hole is not pre-formed in either SplB protease [22] or S1A subfamily protease zymogens [59,60]. In the crystal structure, a water molecule that accepts hydrogen bonds from both amide hydrogen atoms occupies the oxyanion hole of SplD in the absence of a substrate. Water coordination is found in the structures of many members of the S1 family of proteases. It is assumed that a substrate or a canonical inhibitor displaces the water molecule during the initial stages of interaction with the catalytic machinery.

### The primary specificity pocket

Tight spatial fit and specific interactions of the P1 residue side chain in the S1 pocket are the primary determinants of the specificity of the chymotrypsin family of proteases. Accordingly, the S1 specificity pocket of SplD is an easily distinguished cavity that is adjacent to the catalytic triad Ser156 and the oxyanion hole. The cavity is formed by loop 2 (Asp175–Arg183), residues Ala149–Ser155 (including a γ-turn corresponding to an extended loop 1 in chymotrypsin), and a fragment of a β-sheet of one of the barrels (strands Met171–Ser174 and Ser184–Phe185). The pocket is lined with the backbone atoms of these residues, particularly 153–155 and 172–174, and the side chains of Val151, Ser155, Met171, and Ser174. Although polar residues are involved in cavity formation, only their C^β^ atoms are exposed to the surface resulting in a primarily hydrophobic pocket. Therefore, the pocket is comparable to that of human neutrophil elastase (HNE), which shows similar specificity to SplD (Figure S3 in File S1).

### Modeling of substrate binding

**Figure 5:**
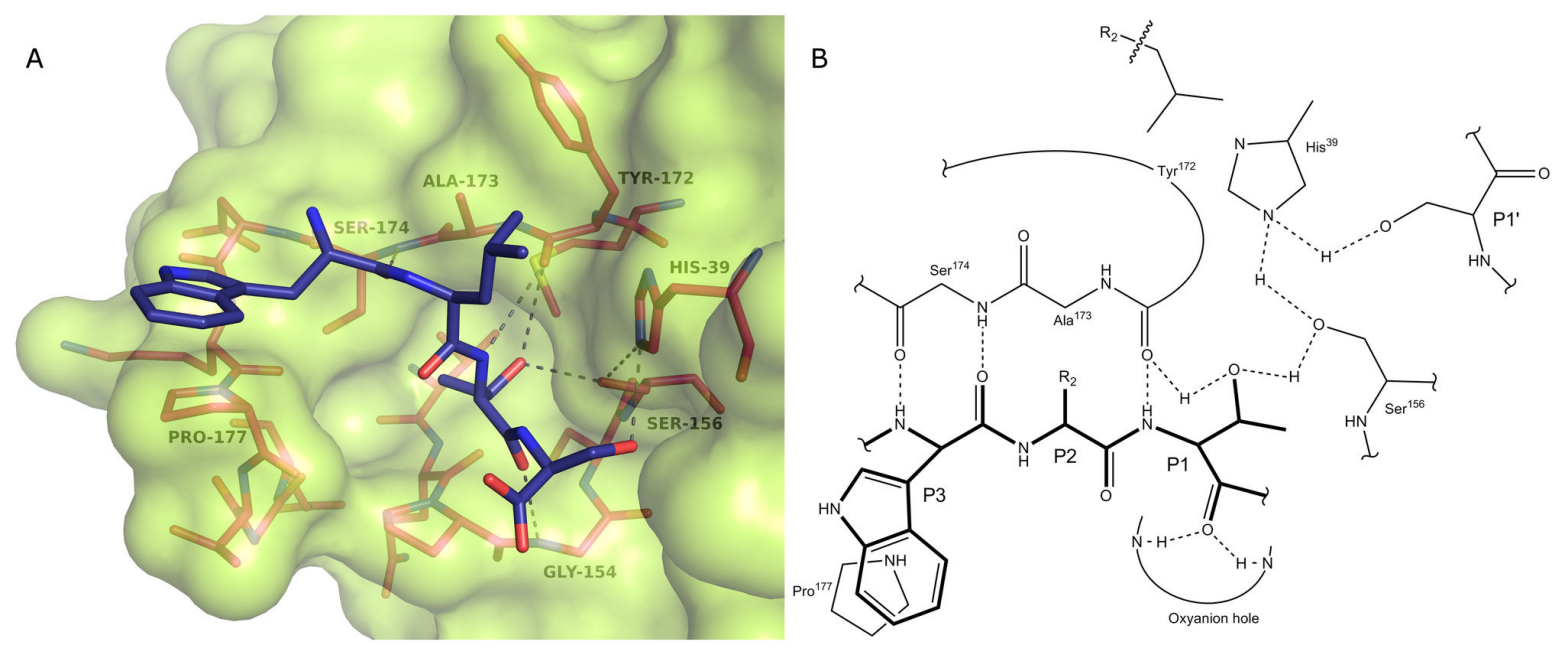
Putative binding mode of the SplD consensus substrate. (A) Residues P4 through P1’ of the consensus substrate (blue) docked to SplD (surface model). (B) Schematic representation of interactions between the consensus substrate (thick lines) and SplD (thin lines). Hydrogen bonds are shown as dotted lines.

Having solved the atomic structure of SplD, we attempted to define the mode of consensus substrate recognition using molecular modeling. The extensive knowledge on substrate / inhibitor binding in the S1 family of proteases [44,45,46] was used to construct the initial model of SplD bound to a peptide spanning residues P3-P1’ of the consensus substrate. Molecular dynamics of the system was simulated over 5 ns and the resulting trajectory was analyzed to in order to define the interaction surface. The predicted interactions are summarized in Figure 5 and Table S4 in File S1. The backbone atoms of the substrate form canonical hydrogen bonds with the enzyme, including two hydrogen bonds between P3 residue and Ser174 and a hydrogen bond between the P1 residue and Tyr172. The side chain of the tryptophan residue at position P3 interacts with Pro177 via van der Waals interactions. The P2 side chain resides in a shallow pocket formed by the side chains of His39, Asp78, and Tyr172. The P1 side chain resides in a canonical S1 pocket. Apart from being stabilized by van der Waals interactions the sidechain O^γ^ of a threonine residue at position P1 forms a hydrogen bond with the sidechain of Ser156. The backbone carbonyl oxygen of the P1 residue resides in the oxyanion hole. The side chain of the P1’ residue alternately forms transient hydrogen bonds with His39 or Ala23. Residues further than P3 and P1’ were not included in the model because of the speculative characteristics of the modeling system in the lack of crystal structures documenting equivalent interactions.

To evaluate the adequacy of our model we tested the influence of residues having model predicted role in substrate recognition on proteolytic activity and specificity of SplD protease. Neither, the predicted S3 subsite distorting Pro177Gly mutant, nor the predicted S2 subsite distorting Tyr172Ala mutant demonstrated detectable activity against ABZ-Trp-Leu-Thr-Ser-ANB-NH2, an efficient substrate of wild type SplD protease (Table 3). At the same time, both mutants retained proteolytic activity against β-casein. This demonstrates that the mutations do not significantly affect the overall protease structure, but have significant effect on substrate recognition, consistent with the predictions of our model. Furthermore, the model predicts an important role of a hydrogen bond between the P1 threonine sidechain and cataltyic triad serine in substrate recognition. This was tested by comparing the kinetics of hydrolysis by wild type SplD of CLIPS derived consensus sequence peptide substrate and an identical substrate where threonine was substituted with valine (Table 3). The affinity (K_m_) of the former substrate was approximately two times stronger than that of the latter being in line with the predictions of our model. Overall, we conclude that the provided model sensibly predicts the interactions responsible for substrate recognition by SplD protease.

## Discussion

### SplD activation

SplD shares 12% amino acid sequence identity with chymotrypsin, the archetype protease of the S1 family [61]. Despite limited similarities in the primary structure, the tertiary structure of the core of both proteins is almost identical, with an RMSD of 2.01 Å over 177 equivalent C_α_ atoms. The majority of proteases of the S1 family belong to subfamily S1A. The activating role of N-terminal processing in this subfamily has been extensively studied. In general, chymotrypsin-like proteases are synthesized as inactive precursors containing N-terminal extensions. Proteolytic processing of a zymogen releases a new N-terminus, which forms a buried salt bridge and induces structural rearrangement of the “activation domain”. The rearrangement orders the S1 site and the oxyanion hole creating an active protease [46]. This activation mechanism is not conserved in the small subfamily S1B, which contains all nine staphylococcal serine proteases and several other enzymes. SplB and possibly SplA require accurate N-terminal processing to achieve full activity, but the structural details of activation differ from those found in the S1A subfamily. Processing is also necessary for V8 protease, but the activation mechanism differs from that of the S1A family and SplB. Epidermolytic toxins possess an additional N-terminal helix and are active without processing. Here, we showed that SplD remains active, despite an artificial N-terminal extension of one (Q-SplD) or two (GS-SplD) amino acids, and the entire GST fusion tag. Despite testing multiple approaches, including enterokinase and factor Xa processing of GST fusion proteins, heterologous production and secretion in a Gram-positive host, or purification from *S. aureus*, we were unable to obtain SplD with an unmodified N-terminus. Therefore, we were unable to compare the activities of modified and native SplD. However, the following findings suggest that exact N-terminal trimming is dispensable for the activation of SplD. First, the k_cat_/K_M_ value for the best identified here SplD substrate (Table 3) was close to that reported for best substrates of other serine proteases [46]. Second, unlike other zymogens of the S1A subfamily, no deformation of the active site was observed in the structure of GS-SplD. 

In SplB, the N-terminal glutamic acid is positioned on the surface of the protease by a network of hydrogen bonds. Comparable interactions maintain the position of the N-terminal Glu in the structure of SplA. In SplA and SplB, the hydrogen bond network involves the side chain and the N-terminal amine group of Glu1. Any modification at the N-terminus, including elongation of the polypeptide chain, abolishes the ability of the N-terminal amine group to form canonical hydrogen bonds and therefore significantly reduces the activity of SplB (SplA has not been tested in this context). Interestingly, we found no differences in the activity of the SplD constructs evaluated in this study, despite the presence of various modifications at the N-terminus. In the structure of GS-SplD, Glu1 has a poorly defined electron density and does not form any hydrogen bonds. Further analyses revealed that this property is not directly due to the artificial N-terminal extension. Instead, SplD appears to have bypassed the requirement for precise N-terminal trimming for its activation. When the N-terminus of SplA or SplB is modeled on the structure of SplD, it becomes clear that the conformation found in SplA and SplB is not supported in SplD by interactions seen in SplA or SplB (Figure S4 in File S1). In particular, the salt bridge between the side chains of Arg112 in SplA (Arg115 in SplB) and Glu1 is not retained in SplD because Pro114 is found at the position equivalent to Arg112 in SplA (Arg115 in SplB). Second, the interaction between loop 2 and the N-terminal amine characteristic of SplA is not supported in SplD. In SplA, this interaction is provided by a hydrogen bond with the side chain of Glu179. In SplB, this region has a poorly defined electron density, but it seems that loop 2 also transiently interacts with the N-terminal amine. Loop 2 is well defined in SplD, but none of the protruding side chains reaches the N-terminal amine, even was it located in a similar position to that in SplA. Although the N-terminal glutamic acid is conserved in all Spl proteases, it seems that the activity of SplD is independent of N-terminal trimming. We speculate that this independence is associated with the natural change of Arg112 (Arg115) to proline. In this context, it will be interesting to determine the effects of N-terminus processing on the proteolytic activity of SplE and SplF, which contain arginine and proline, respectively, at the position equivalent to Arg115 of SplB.

The proteolytic activity of N-terminally extended SplD explains the problems encountered trying to induce efficient heterologous expression. Compared with the GST-SplA, GST-SplB, and GST-SplC fusion proteins, which were efficiently expressed in *E. coli*, GST-SplD required extensive optimization, but the yields were still low, probably because of a deleterious effect of overexpressing an active protease.

### Primary specificity pocket

The catalysis mechanism and structural basis of substrate recognition in the S1 family are among the most thoroughly characterized aspects of enzymology. However, it is still difficult to predict the substrate specificity of a novel member of the family using the amino acid sequence alone, which means that experimental characterization is necessary. Here, we showed that the activity of SplD is limited to substrates containing alanine, valine, leucine, isoleucine, serine, and threonine at position P1. Glutamine is also accepted, but such substrates are cleaved with very low efficiency. Other residues are excluded from the P1 site. Among chymotrypsin-like proteases with known X-ray structures, one with a comparable substrate specificity is HNE (Figure S3 in File S1) [62,63]. 

The S1 cavity of SplD and HNE is an interesting example of convergent evolution that led to markedly different structural organizations of the S1 pocket, but with comparable van der Waals surfaces. The disposition of the main chain around the cavity is almost identical in both structures, except for loop 2. In HNE, the loop does not close the south-western part of the pocket as closely as that observed in SplD. However, in HNE, the resulting empty space is occupied by the side chain of Phe192 and the molecular surfaces of both pockets are comparable. Another significant difference is located at the northern bottom part of the pocket. The bottom of this pocket is lined with the side chain of Met171 in SplD and with the side chain of Ala213 (equivalent to Met171 in SplD) and Phe228 in HNE. Despite this difference, the van der Waals surface of this section of the pocket is similar in both enzymes because the positions of the side chain atoms of Met171 correspond to pocket exposed atoms of Ala213 and Phe228. Further differences were found in the southern and northern bottom sides of the pocket that are lined respectively by the side chains of Ser155 and Ser184 in SplD, and of Asp194 and Asp226 in HNE. However, because only the C^β^ atoms are exposed to the surface of the pocket and the positions of these atoms are equivalent in both structures, the shapes of the pockets are similar. Therefore, despite the different compositions of the residues that form the S1 pocket between SplD and HNE, the overall physicochemical properties of the cavity are similar, which explains the similar substrate specificities of both enzymes.

### Substrate recognition model

Having solved the crystal structure of SplD, we sought to determine the likely binding mode of the consensus sequence substrate. To achieve this, we performed homology modeling and molecular dynamics analyses. Based on the extensive characterization of the substrate/inhibitor–protease interaction in the S1A family, the interactions at the S3-S1’ pockets were likely to be modeled correctly. Modeling of the interactions further than P3 and P1’ was not attempted because of limited reference structural information. The validity of the proposed model was verified using mutants of residues of predicted importance in substrate recognition. These mutants demonstrated no activity against specific substrate of wild type SplD, but retained activity against nonspecific protein suggesting a shift in substrate specificity of the mutants, in favor of model predictions. Below, the feasibility of the predicted model is further verified against the available structural data. Interestingly, molecular modeling suggested that the side chain of threonine at P1 is stabilized in the S1 binding pocket of SplD by an energetically favorable hydrogen bond between threonine O^γ^ and the side chain hydroxyl of catalytic triad Ser156, the central residue in the catalytic process. PDB was queried to determine whether this interaction was observed in previous crystallographic studies. The structures of streptogrisin B in a complex with the Thr18 variant of the third domain of turkey ovomucoid inhibitor (PDB ID 1CT2) [64] and an inhibitory complex between α-lytic protease and its proregion (PDB ID 4PRO) [65], both contain a hydrogen bond between O^γ^ of threonine at position P1 and the side chain of catalytic triad serine, as observed in our model.

Unlike the S1 specificity pocket, the S2 and S3 subsites of SplD do not form pronounced cavities. Leucine is often selected at the S2 subsite by proteases of the chymotrypsin family, resulting in a wealth of structural information. The interactions formed at S2 subsite by the side chain of leucine at position P2 are clearly defined in the complex between chymotrypsin and N-Ac-Leu-Phe-CHO (PDB ID 1GGD), between streptogrisin A and chymostatin (PDB ID 1SGC), and in many other structures. In most of those enzymes, the P2 pocket is composed of a pronounced cavity formed by the side chain of catalytic triad histidine, loop C, and loop 3 in some enzymes. In SplD, loops C and 3 are short and are unable to support classical S2 pocket formation. Instead, the P2 binding site of SplD is a shallow patch formed by the side chains of His39, Asp78, and Tyr172, rather than a cavity. Elements of the S2 pocket provided by loop C in chymotrypsin are missing in SplD. Nevertheless, despite the simpler pocket structure of SplD, it is still able to support the limited specificity at the S2 subsite, as demonstrated by the results of our high-throughput substrate screening studies.

The interaction between SplD and the backbone of the P3 substrate residue was modeled according to that of canonical inhibitors. In this model, the two characteristic hydrogen bonds (β-sheet-like bonds with Ser174) were preserved throughout the simulation (Table S4 in File S1). Side chain interactions at the S3 subsite are rarely defined in proteases from the chymotrypsin family, and we are unaware of any structures that could help to validate our predicted model. According to our model, the substrate specificity at the S3 subsite is driven by a “stacked-like” interaction between the P3 tryptophan indole moiety and Pro177 and this prediction is favored by the properties of Pro177Gly mutant. The contribution of such bonds to binding energy was previously estimated to be ~7 kcal mol^-1^ [66].

### Potential physiological role of SplD

Although the role of *spl* operon proteases in staphylococcal physiology remains unknown, some proteases may be involved in the virulence of this bacterium. The current study did not address this question, but provides important topics for further investigation. Having determined the substrate preference of SplD protease, we analyzed the human proteome for likely targets. Of all of the putative SplD substrates identified (Table S5 in File S1), several members of the olfactory receptor family are particularly noteworthy. Olfactory receptors are expressed in the nares, the primary colonization niche of *S. aureus*. Because olfactory receptors are transmembrane proteins, their extracellular moieties may be directly accessible to the Spl proteases. Moreover, putative cleavage sites in other members of the olfactory receptor family were identified based on the substrate specificities of SplA [23] and SplB [22]. Therefore, it is tempting to speculate that the hydrolysis of olfactory receptors by Spl proteases contributes to staphylococcal persistence in the nares, although no experimental evidence is currently available to support this hypothesis.

An important aspect of *in silico* substrate prediction is the consistency between the physiological substrates and the specificity determined in high-throughput assays. Physiologically, the selection of substrates is determined by the polypeptide sequence around the hydrolyzed peptide bond and other factors. More abundant substrates may be kinetically favored. Steric hindrance, compartmentalization, and the effect of other factors should also be considered. Some proteases with well-characterized physiological targets, *in vitro*, in high-throughput screens, show preference for sequences that are similar, although not identical to those recognized in the target substrate [32] which complicates *in silico* prediction of targets. However, it is certain that, in the S1 family, the S1 subsite shows high specificity. In this context, the available data on the substrate specificity of Spl proteases suggests that they exhibit cooperative activity. SplA hydrolyzes substrates with tyrosine/phenylalanine in the P1 position (chymotrypsin-like activity), SplB cleaves substrates after glutamine, asparagine, or aspartic acid (glutamyl endopeptidase-like activity), and SplD selects threonine, isoleucine, leucine, valine, alanine, and serine at P1 (elastase-like activity). Therefore, it will be intriguing to determine whether the remaining three Spl proteases exhibit activity that is complementary to SplA, SplB, and SplD. The fact that all six *spl* genes are co-transcribed [18] further strengthens the possibility of mutual activities. Cooperation of proteases with complementary substrate specificities has been extensively characterized for the digestive enzymes trypsin, chymotrypsin, and pancreatic elastase. Whether the Spl proteases play a specific role in targeting a limited number of specific substrates, constitute a general digestive system, or have another undocumented role, remains to be determined in future studies.

## Supporting Information

File S1 **Figure S1** , Overall fold of SplD protease. **Figure** **S2**, Interpretation of the orientation of the sidechain of catalytic triad Ser156 in the crystal structure of SplD. **Figure** **S3**, Comparison of substrate specificities of SplD and HNE proteases. Figure **S4**, Disposition of the N-terminal glutamic acid residue in the structures of SplA, SplB and GS-SplD proteases. **Note** **S1**, Ambiguity of P(n) and P(n)’ subsite specificity determination in fluorescence quenched LSTS assay. **Note** **S2**, Conformation of the catalytic triad serine in the crystal structure of SplD protease. **Table** **S1**, synthetic substrates tested for hydrolysis by SplD protease. **Table** **S2**, SplD substrate specificity determined using CLIPS. **Table** **S3**, Average main chain angles of the residues forming the oxyanion hole. **Table** **S4**, Predictions of the in silico model of SplD interaction with consensus substrate. **Table** **S5**, Potential physiological substrates of SplD protease.
(DOC)
