# Supplementary material for: Biochemical and Structural Characterization of SplD Protease from Staphylococcus aureus
Source: PLoS One. 2013 Oct 9;8(10):e76812. doi: 10.1371/journal.pone.0076812 (PMC3793935; doi:10.1371/journal.pone.0076812)
Supplement: File S1 — Figure S1 , Overall fold of SplD protease. Figure S2, Interpretation of the orientation of the sidechain of catalytic triad Ser156 in the crystal structure of SplD. Figure S3, Comparison of substrate specificities of SplD and HNE proteases. Figure S4, Disposition of the N-terminal glutamic acid residue in the structures of SplA, SplB and GS-SplD proteases. Note S1, Ambiguity of P(n) and P(n)’ subsite specificity determination in fluorescence quenched LSTS assay. Note S2, Conformation of the catalytic triad serine in the crystal structure of SplD protease. Table S1, synthetic substrates tested for hydrolysis by SplD protease. Table S2, SplD substrate specificity determined using CLIPS. Table S3, Average main chain angles of the residues forming the oxyanion hole. Table S4, Predictions of the in silico model of SplD interaction with consensus substrate. Table S5, Potential physiological substrates of SplD protease. (DOC) [file pone.0076812.s001.doc]

**SUPPORTING INFORMATION**

**Table of contents:**

**1. Note S1. Ambiguity of P(n) and P(n)’ subsite specificity determination in fluorescence quenched LSTS assay**

**2. Note S2. Conformation of the catalytic triad serine in the crystal structure of SplD protease**

**3. Supplementary figures:**

**Figure S1. Overall fold of SplD protease.**

**Figure S2. Interpretation of the orientation of the sidechain of catalytic triad Ser156 in the crystal structure of SplD.**

**Figure S3. Comparison of substrate specificities of SplD and HNE proteases.**

**Figure S4. Disposition of the N-terminal glutamic acid residue in the structures of SplA, SplB and GS-SplD proteases.**

**4. Supplementary tables:**

**Table S1. Synthetic substrates tested for hydrolysis by SplD protease.**

**Table S2. SplD substrate specificity determined using CLIPS.**

**Table S3. Average main chain angles of the residues forming the oxyanion hole.**

**Table S4. Predictions of the *in silico* model of SplD interaction with consensus substrate.**

**Table S5. Potential physiological substrates of SplD protease.**

**5. References**

**1. Note S1. Ambiguity of P(n) and P(n)’ subsite specificity determination in fluorescence quenched LSTS assay**

Selection of the LSTS library of a general structure ABZ-X4-X3-X2-X1-ANB-NH_2_ was performed in two detection modes – absorbance and fluorescence. In the absorbance selection mode the library positions X4-X1 correspond to P4-P1 positions of the substrate since only cleavage of the X1-↓-ANB-NH_2_ bond releases a colored product while hydrolysis of other peptide bonds inside the library (i.e. X4-↓-X3, X3-↓-X2 or X2-↓-X1) does not result in the increase of absorbance. Differently, when the release of quenched fluorescence is monitored cleavage of any bond within the substrate (i.e. X4-↓-X3, X3-↓-X2, X2-↓-X1, or X1-↓-ANB-NH_2_) results in fluorescence increase. Therefore selection of the X4 library in the fluorescence detection mode reflects cumulative hydrolysis at all mentioned positions. It can be assumed that while X4 is fixed with particular residue the hydrolysis of the X3 library is partially directed by the fixed residue towards hydrolysis of a particular peptide bond. However, it may not be excluded that other bonds are also concomitantly hydrolyzed, the extent of which is not determined during the experiment. It is further assumed that fixing of X4 and X3 positions has a stronger effect on directing the hydrolysis of X2 library towards particular peptide bond, but still the extent of this effect is not determined during the experiment. Only the selection of the last (X1) library almost unambiguously determines substrate specificity at X1 position (which can reflect any of the substrate positions from P1 to P3’; P3’ in this study) assuming that the cleavage site inside the substrate is already established by the fixed residues (X4, X3 and X2). However, this data may still reflect cumulative effect of cleavage between X1-↓-ANB-NH_2_ and possibly at other sites. Only when all libraries are selected, thus determined best substrate is resynthesized and the cleavage site is determined using mass spectrometry. In the particular case of SplD protease the substrate is hydrolyzed between X4-↓-X3 residues.

Overall, the selection in the absorbance mode is best suited for determining the P4-P1 substrate preference, but not necessarily to obtain an efficient substrate since P(n)’ positions are not optimized. Conversely, selection in the fluorescence mode allows to determine most efficient substrates of a general structure of ABZ-X4-X3-X2-X1-ANB-NH_2_ allowing for optimization of both the primed as well as non-primed positions, but is poorly suited for resolving the absolute specificity at particular subsites.

**2. Note S2. Conformation of the catalytic triad serine in the crystal structure of SplD protease**

In the proteases of family S1 the side chain of catalytic triad serine is commonly found in *gauche+* rotamer. Such orientation supports canonical hydrogen bond with catalytic triad histidine, a prerequisite for the activation of catalytic triad serine O^γ^ atom supporting nucleophilic attack on the carbonyl carbon of the scissile peptide bond .

Our data demonstrate that the catalytic triad serine of SplD protease adopts *gauche+*(χ_1_ ~ -60°), *gauche-* (χ_1_ ~ +60°) and *trans* (χ_1_ ~ +180°) side chain rotamers (Figure S2). Those orientations represent either the time-averaged structure within the unit cell or the average of different conformations present within different unit cells comprising the crystal lattice which can not be distinguished based on the collected data. Since SplD readily demonstrates proteolytic activity in the crystallization buffer the observed conformations are characteristic of an active enzyme rather than reflecting an inactive form induced by crystallization conditions. Most probably the rotamers interchange and possibly the canonical *gauche+* orientation is stabilized through substrate binding. A comparable situation was previously observed in the structures of coagulation factor Xa (1C5M; ) and kallikrein 1 (1SPJ; ), both of which are active serine proteases. For example in kallikrein 1, catalytic Ser195 adopts *gauche+* (χ_1_ ~ -60°) and *gauche-* (χ_1_ ~ +60°) conformations. It was postulated that the binding of a P1 residue serves to place catalytic serine in a productive orientation ).

**3. Supplementary Figures**


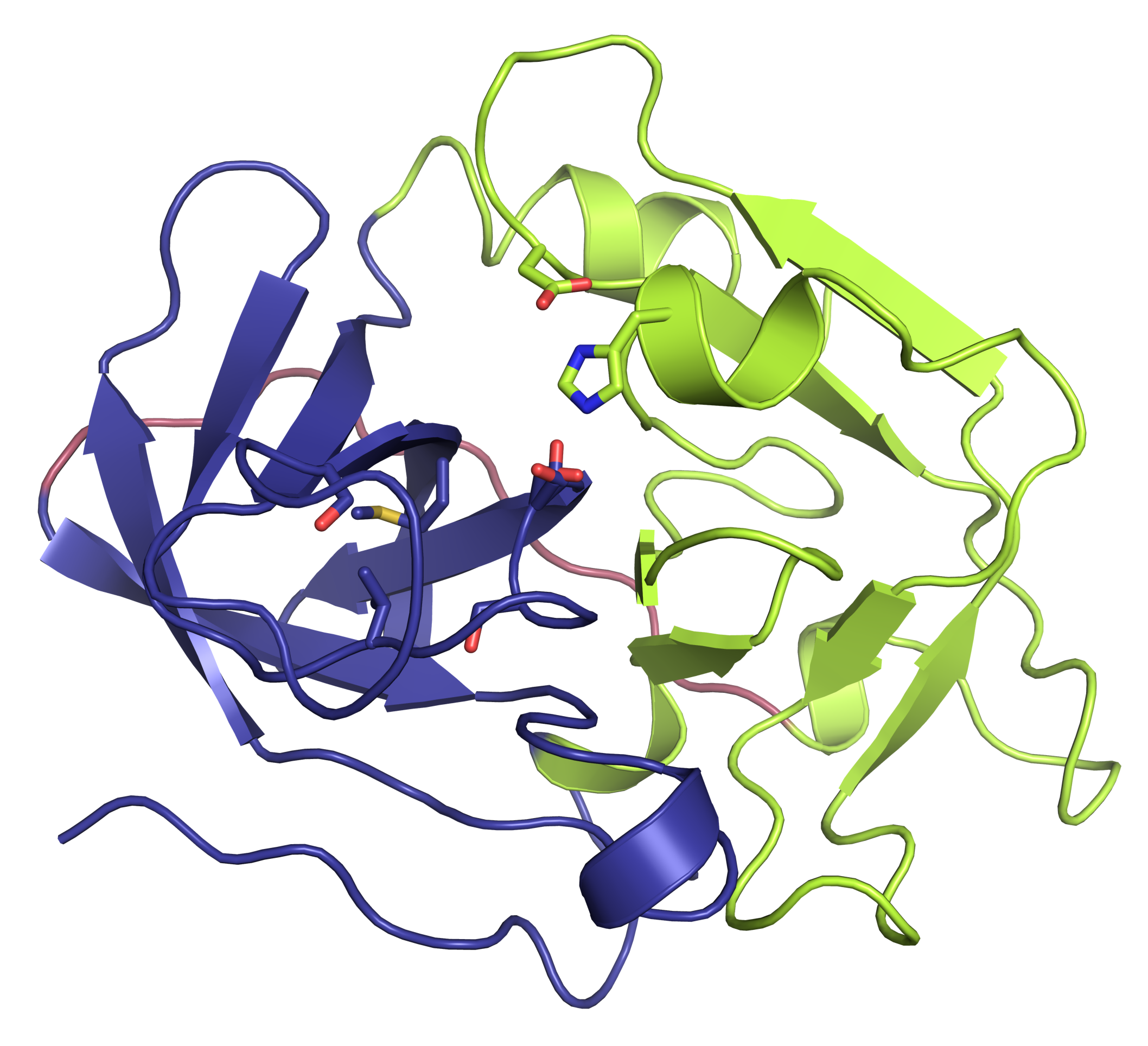


**Figure S1: Overall fold of SplD protease. SplD shows a typical chymotrypsin-like fold. Domains I and II are shown in limon and blue, respectively. A connecting linker of 14 residues (Thr100-Glu113) is depicted in pink. The side chains of catalytic triad residues His39, Asp78, Ser156 and the residues lining the S1 pocket (Val151, Ser155, Met171, Ser174) are drawn in stick representation. The side chain of catalytic Ser156 adopts three alternative conformations in the SplD structure.**


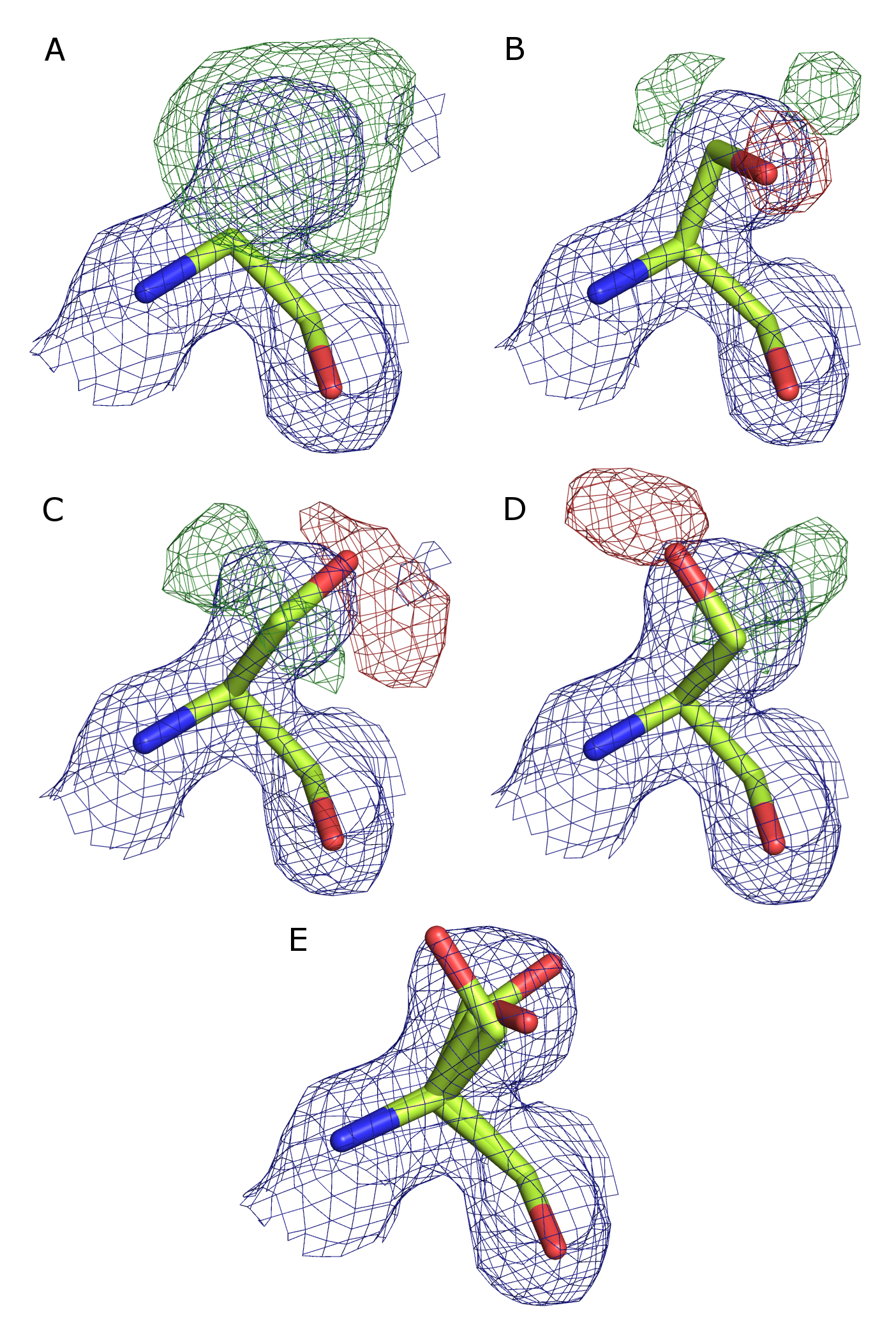


**Figure S2. Interpretation of the orientation of the sidechain of catalytic triad Ser156 in the crystal structure of SplD. Electron denisty maps encompassing Ser156 residue calculated using models containing different orientations of the residue are shown. (A) side chain of Ser156 omitted; (B) side chain in *gauche*- conformation; (C) side chain in *trans* conformation; (D) side chain in *gauche*+ conformation; (E) split side chain in alternative *gauche*+, *gauche*- and *trans* conformations. 2Fo-Fc maps were contoured at 1σ (blue) and Fo-Fc maps were contoured at 2.5σ (positive peaks – green; negative peaks – red).**

**
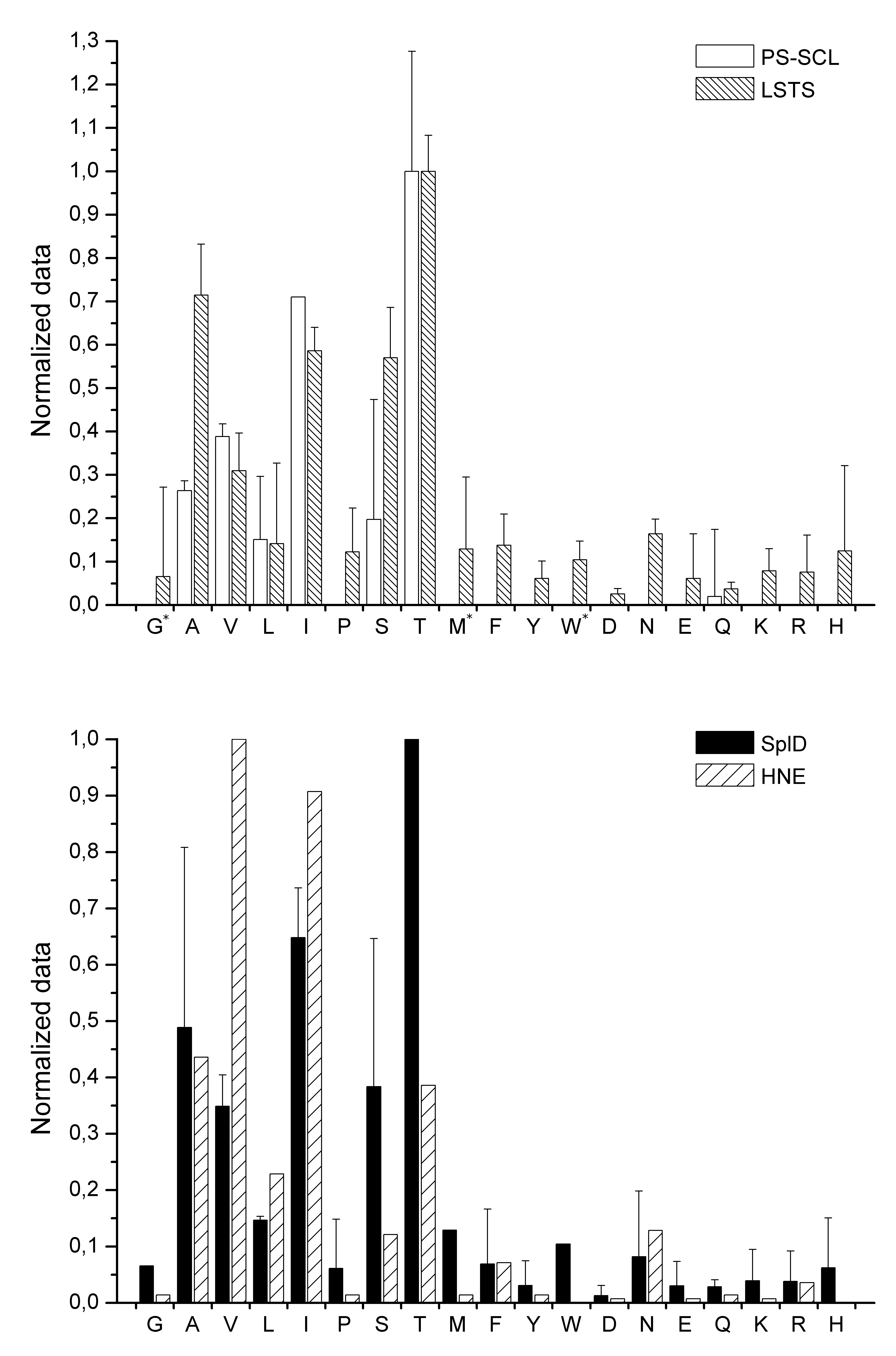
**

**Figure S3. Comparison of substrate specificities of SplD and HNE proteases. (A) P1 substrate specificity of SplD determined using PS-SLC assay (white bars) and LSTS assay in the absorbtion detection mode (shaded bars). Asterisks indicate substrate residues not assessed in PS-SLC method (residues absent in the PS-SLC substrate sublibraries). (B) P1 substrate specificity of SplD exemplified as an average of specificities determined using PS-SLC and LSTS from panel A (black bars). P1 substrate specificity of HNE (shaded bars) determined as an average over all 477 HNE substrates collected in the MEROPS database (http://merops.sanger.ac.uk/). Note that such presented substrate specificity of HNE is not an effect of a single high throughput experiment where all the substrates are equally represented in the initial pool, but rather is most probably biased towards certain substrates in differet adapted experimental approaches. At the same time a large number of evaluated substrates derived from different studies most probably averages the above described bias to a certain extent.**

**
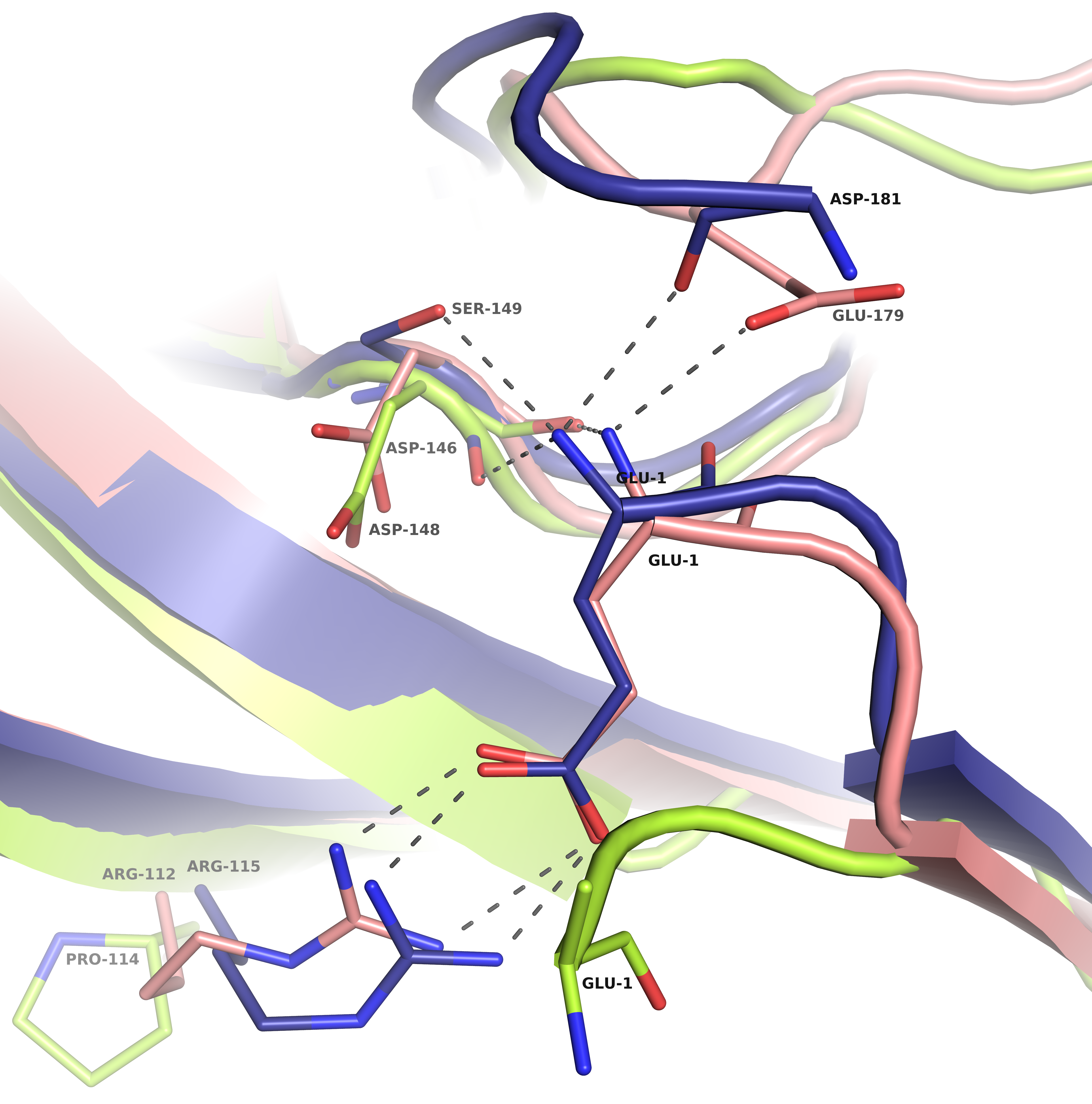
**

**Figure S4. Disposition of the N-terminal glutamic acid residue in the structures of SplA, SplB and
GS-SplD proteases. The N-terminal glutamic acid of SplA (pink) and SplB (blue) forms as a characteristic network of hydrogen bonds – a salt bridge with Arg112 (SplA) or equivalent Arg115 (SplB) and hydrogen bonds between the N-terminal amine and the backbone oxygen of Asp146 and O^ε^ of Glu179 (SplA) or backbone oxygen and Oγ of Ser149 and the backbone oxygen of Asp181 (SplB). The described hydrogen bond network is absent in the structure of GS-SplD (limon) where Pro114 is found at the position corresponding to Arg112 (SplA) or Arg115 (SplB) and the artificial N-terminal extension precludes formation of hydrogen bonds of the amine group of Glu1. (The side chain of Glu1 and the residues Ser0 and Gly(-1) of SplD are not defined by electron density and therefore not shown in the figure).**

**4. Supplementary Tables**

**Table S1. Synthetic substrates tested for hydrolysis by SplD protease.**

| **Substrate** | **Activity** | **Substrate** | **Activity** |
| --- | --- | --- | --- |
| Leu-Leu-pNA | **-** | Boc-Leu-Arg-Arg-MCA | **-** |
| Pyr-Phe-Leu-pNA | **-** | Suc-Leu-Leu-Val-Tyr-MCA | **-** |
| Suc-Ala-Ala-Pro-Leu-pNA | **+** | Boc-Leu-Gly-Arg-MCA | **-** |
| Z-Gly-Gly-Leu-pNA | **-** | Boc-Leu-Thr-Arg-MCA | **-** |
| Benzoyl-Val-Gly-Arg-pNA | **-** | Boc-Val-Leu-Lys-MCA | **-** |
| Boc-Val-Leu-Gly-Arg-pNA | **-** | Suc-Gly-Pro-Leu-Gly-Pro-AMC | **-** |
| D-Val-Leu-Lys-pNA | **-** | H-Met-Leu-AMC | **-** |
| Gly-Pro-pNA | **-** | Suc-Ala-Ala-Ala-AMC | **-** |
| H-Gly-Arg-pNA | **-** | H-Leu-AMC | **-** |
| N-p-Tosyl-Gly-Pro-Lys-pNA | **-** | Benzoyl-Phe-Val-Arg-AMC | **-** |
| Suc-Ala-Ala-Pro-Asp-pNA | **-** | Z-Gly-Gly-Leu-AMC | **-** |
| Suc-Ala-Ala-Pro-Glu-pNA | **-** | Z-Leu-Leu-Glu-AMC | **-** |
| Suc-Val-Pro-Phe-pNA | **-** | Z-Leu-Leu-Arg-AMC | **-** |
| Boc-Asp(OBzl)-Pro-Arg-MCA | **-** | Boc-Val-Pro-Arg-AMC | **-** |
| Boc-Gln-Arg-Arg-MCA | **-** | Abz-Glu-Gly-Ile-Gly-Thr-Ser-Arg-Pro-Lys(Dnp)-Asp-OH | **-** |
| Boc-Gly-Arg-Arg-MCA | **-** | Abz-Glu-Ala-Leu-Gly-Thr-Ser-Pro-Arg-Lys(Dnp)-Asp-OH | **-** |
| Boc-Gly-Lys-Arg-MCA | **-** |  |  |

**Table S2. Aligned amino acid sequences selected as targets of SplD protease using CLIPS**

In the eCPX protein used for selection the variable substrate sequence (eight consecutive amino acids; capital letters) was flanked with a constant sequence of gsgqgg-(X)_8_-ggsggs (small letters). Dashed line separates sequences obtained from two subsequent rounds of enrichment. Obtained consensus sequence recognized by SplD protease is shown at the bottom of the table. Residues corresponding to the determined consensus are highlighted bold. The table presents of the same experiment as Table 1 of the manuscript save only it extends over the entire variable region of each substrate and depicts sequences derived of all selected clones including those not included in the final alignment presented in Table 1 due to their ambigous placement relative to the consensus (omited sequences are indicated with an asterisks).

|  |  | g | P | R | L | **Y** | **P** | **M** | **S** | S | g |  |  |  | * |
| --- | --- | --- | --- | --- | --- | --- | --- | --- | --- | --- | --- | --- | --- | --- | --- |
|  |  | g | Y | R | L | **F** | **P** | **V** | **S** | A | G |  |  |  |  |
|  |  |  | g | W | V | **Y** | **P** | **V** | **D** | M | H | g |  |  |  |
|  |  | g | S | R | V | **Y** | S | **T** | **S** | Y | g |  |  |  |  |
|  |  |  |  | g | **R** | **Y** | W | **T** | **S** | G | M | L | g |  |  |
|  |  |  |  | g | **R** | **Y** | **P** | **T** | **S** | W | Y | P | g |  |  |
|  |  | g | P | P | **R** | **F** | Q | **M** | **S** | S | g |  |  |  | * |
|  |  |  | g | T | **R** | **W** | **L** | **L** | **S** | S | P | g |  |  |  |
|  |  |  |  | g | **R** | **W** | **P** | **T** | **S** | K | P | L | g |  |  |
|  |  |  |  | g | **R** | H | **P** | **M** | G | F | S | L | g |  | * |
|  | g | Y | R | I | T | H | **I** | **L** | **S** | g |  |  |  |  |  |
|  |  | g | g | P | **R** | **Y** | **L** | **L** | T | S | T | g |  |  |  |
| g | P | G | S | V | **R** | **Y** | **L** | **M** | g |  |  |  |  |  | * |
|  |  |  | g | R | Q | **W** | **L** | **L** | A | S | L | g |  |  |  |
|  |  |  | g | R | Q | **Y** | **L** | **L** | G | A | I | g |  |  |  |
|  |  | g | F | A | **R** | H | **P** | A | F | g |  |  |  |  | * |
|  |  | g | S | I | **R** | **Y** | **L** | **T** | **S** | L | g | g |  |  |  |
|  |  |  |  | g | **R** | **Y** | **L** | **T** | G | L | S | g |  |  |  |
|  |  |  | g | G | **R** | **Y** | **L** | **T** | G | L | S | g |  |  |  |
|  |  |  |  | g | **R** | **Y** | **P** | **T** | **S** | W | Y | P | P | g |  |
|  |  | g | P | P | **R** | **F** | Q | **M** | **S** | S | g |  |  |  | * |
|  | g | R | W | P | M | **W** | Q | **V** | **S** | g |  |  |  |  | * |
|  |  |  | g | K | T | **Y** | **P** | **I** | **S** | S | T | g |  |  |  |
|  |  |  | g | R | V | **Y** | G | **I** | **S** | S | F | g |  |  |  |
|  |  | g | Y | R | L | **F** | **P** | **V** | **S** | A | g |  |  |  |  |
|  |  |  | g | R | L | **Y** | **P** | **I** | **S** | A | Y | g |  |  |  |
|  |  |  |  |  | **R** | **Y/W/F** | **P/L** | **T/L/M/V/I** | **S** |  |  |  |  |  |  |

**Table S3. Average main chain angles of the residues forming the oxyanion hole.**

The ϕ and ψ main chain angels are compared in serine proteases of family S1 having canonical and distorted oxyanion hole.

|  | Residue n-3 relative to catalytic triad serine (n) | ϕ [º] | ψ[º] | Residue n-2 relative to catalytic triad serine (n) | ϕ[º] | ψ[º] | PDB ID |
| --- | --- | --- | --- | --- | --- | --- | --- |
| Canonical conformation of the oxyanion hole | | | | | | | |
| SplD | Pro153 | -42 | 135 | Gly154 | 153 | -32 | 4ink |
| Chymotrypsin | Met192 | -62 | 121 | Gly193 | 167 | -14 | 1yph |
| V8 protease | Gly166 | -50 | 130 | Gly167 | 150 | -29 | 1qy6 |
| Distorted oxyanion hole | | | | | | | |
| ETA | Pro192 | 138 | -41 | Gly193 | 158 | -21 | 1exf |
| SplB | Ser 154 | 144 | -36 | Gly155 | 162 | -19 | 2vid |
| FVIIa | Lys192 | 130 | -50 | Gly193 | 167 | -13 | 1wtg |

**Table S4. Predictions of the *in silico* model of SplD interaction with consensus substrate.**

Summary of hydrogen bonds formed between the protease and the substrate during 5ns molecular dynamics simulation. Contribution of particular hydrogen bonds is expressed as the percent of time they are present during the entire simulation (bonds present less than 20% of total simulation time are not presented). The hydrogen bonded substrate atoms are marked as donors (^d^) or acceptors (^a^).

| Substrate position | H-**WLTS**-OH | |
| --- | --- | --- |
| P3 (Trp) | O^a^ – **Ser174** N  N^d^ – **Ser174** O | **98.4%**  **88.2%** |
| P2 (Leu) | –––––– |  |
| P1 (Thr) | OG1^d^ – **Ser156** OG  O^a^ – **Gly154** N  N^d^ – **Tyr172** O  O^a^ – **Ser156** N | **86.8%**  **67.1%**  **66.0%**  **33.2%** |
| P1’ (Ser) | OG^d^ – **His39** NE2  OG^d^ – **Ala23** O | **26.3%**  **20.7%** |

**Table S5. Potential physiological substrates of SplD protease**

Uniprot database was queried with the WLLS sequence, an SplD protease cleavage site consensus variant. Shown are all 100 unique hits found in the proteome of *Homo sapiens* (taxid: 9606), and all 4 unique hits within the proteome of *Staphylococcus aureus* (taxid: 1280).

| Accession number | Full protein name | Cellular compartment | Cellular compartment in which the region of potential cleavage is present | Protein length [aa] and position of WLLS sequence |
| --- | --- | --- | --- | --- |
| *Homo sapiens*, taxid: 9606 | | | | |
| **Q9NRX6** | **Protein kish-B** | Golgi apparatus membrane | Extracellular | 74 aa 33-36 |
| **A8MTW9** | Putative uncharacterized protein | Uncertain | Uncertain | 85 aa 64-67 |
| **Q5VUM1** | UPF0369 protein C6orf57 | Extracellular | Potential signal peptide | 108 aa 8-11 |
| **Q6RUI8** | Uncharacterized protein C19orf48 | Uncertain | Uncertain | 117 aa 42-45 |
| Q96KH6 | Uncharacterized protein C18orf12 | Uncertain | Uncertain | 178 aa 70-73 |
| A4D250 | B-cell acute lymphoblastic leukemia-expressed protein | Uncertain | Uncertain | 179 aa 43-46 |
| Q99942 | E3 ubiquitin-protein ligase RNF5 | Plasma membrane  Mitochondrion membrane  ER membrane | Membrane | 180 aa 176-179 |
| Q86X19 | Transmembrane protein 17 | [Cell membrane](http://www.uniprot.org/keywords/KW-1003) [Cell projection](http://www.uniprot.org/keywords/KW-0966) [Cilium](http://www.uniprot.org/keywords/KW-0969) [Membrane](http://www.uniprot.org/keywords/KW-0472) | Membrane | 198 aa 114-117 |
| Q9H902 | Receptor expression-enhancing protein 1 | Mitochondrion membrane  ER membrane | Uncertain | 201 aa 75-78 |
| A0PJX2 | Uncharacterized protein C20orf118 | Uncertain | Uncertain | 215 aa 212-215 |
| O96004 | Heart- and neural crest derivatives-expressed protein 1 | Nucleus | Nucleus | 215 aa 45-48 |
| Q9NZC2 | Triggering receptor expressed on myeloid cells 2 | Cell membrane | Extracellular | 230 aa 70-73 |
| Q15125 | 3-beta-hydroxysteroid-Delta(8),Delta(7)-isomerase | ER membrane | Membrane | 230 aa 47-50 |
| Q9BVC6 | Transmembrane protein 109 | [Endoplasmic reticulum](http://www.uniprot.org/keywords/KW-0256) [Membrane](http://www.uniprot.org/keywords/KW-0472) [Nucleus](http://www.uniprot.org/keywords/KW-0539) [Sarcoplasmic reticulum](http://www.uniprot.org/keywords/KW-0703) | Membrane | 243 aa 143-146 |
| Q9BRK0 | Receptor expression-enhancing protein 2 | Membrane | Uncertain | 252 aa 75-78 |
| Q6NUK4 | Receptor expression-enhancing protein 3 | Membrane | Membrane | 255 aa 75-78 |
| Q9H6H4 | Receptor expression-enhancing protein 4 | Membrane | Uncertain | 257 aa 75-78 |
| P15328 | Folate receptor alpha | Cell membrane | Propeptide (235-257) removed in mature form | 257 aa 254-257 |
| Q6GPI1 | Chymotrypsinogen B2 | Extracellular | Signal peptide (1-18) | 263 aa 5-8 |
| P17538 | Chymotrypsinogen B | Extracellular | Signal peptide (1-18) | 263 aa 5-8 |
| O43819 | Protein SCO2 homolog, Mitochondrial | Mitochondrion | Potential mitochondrial transit peptide (1-41) | 266 aa 36-39 |
| Q9Y275 | Tumor necrosis factor ligand superfamily member 13B | Cell membrane | Extracellular | 285 aa 168-171 |
| P49675 | Steroidogenic acute regulatory protein, Mitochondrial | Mitochondrion matrix | Mitochondrion matrix | 285 aa 241-244 |
| P00491 | Purine nucleoside phosphorylase | Cytoplasm | Cytoplasm | 289 aa 16-19 |
| P58182 | Olfactory receptor 12D2 | Cell membrane | Membrane | 307 aa 195-198 |
| Q96T55 | Potassium channel subfamily K member 16 | Membrane | Cytoplasm | 309 aa 265-268 |
| Q86SM8 | Mas-related G-protein coupled receptor member E | Cell membrane | Cytoplasm | 301 aa 46-49 |
| Q13606 | Olfactory receptor 5I1 | Cell membrane | Membrane | 314 aa 199-202 |
| Q9UGF7 | Olfactory receptor 12D3 | Cell membrane | Membrane | 316 aa 195-198 |
| Q1HG44 | Dual oxidase maturation factor 2 | ER membrane | Membrane | 320 aa 191-194 |
| Q8NEL0 | Coiled-coil domain-containing protein 54 | Uncertain | Uncertain | 328 aa 274-277 |
| Q9BXJ3 | Complement C1q tumor necrosis factor-related protein 4 | Extracellular | Extracellular | 329 aa 285-288 |
| O75031 | Heat shock factor 2-binding protein | Cytoplasm | Cytoplasm | 334 aa 262-625 |
| Q03395 | Rod outer segment membrane protein 1 | Membrane | Membrane | 351 aa 22-25 |
| Q6ZN32 | ETS translocation variant 3-like protein | Nucleus | Nucleus | 361 aa 301-304 |
| P46092 | C-C chemokine receptor type 10 | Cell membrane | Membrane | 362 aa 166-169 |
| Q9NS00 | Glycoprotein-N-acetylgalactosamine 3-beta-galactosyltransferase 1 | Membrane | Uncertain | 363 aa 181-184 |
| O75593 | Forkhead box protein H1 | Nucleus | Nucleus | 365 aa 358-361 |
| Q8NHU3 | Phosphatidylcholine:ceramide cholinephosphotransferase 2 | Cell membrane  Golgi apparatus membrane | Membrane | 365 aa 256-259 |
| P41145 | Kappa-type opioid receptor | Cell membrane | Membrane | 380 aa 183-186 |
| Q86T26 | Transmembrane protease serine 11B | Membrane | Extracellular | 416 aa 219-222 |
| Q86VZ5 | Phosphatidylcholine:ceramide cholinephosphotransferase 1 | Golgi apparatus membrane | Membrane | 419 aa 318-321 |
| Q9Y276 | Mitochondrial chaperone BCS1 | Mitochondrion inner membrane | Mitochondrial matrix | 419 aa 62-65 |
| Q7RTY0 | Monocarboxylate transporter 13 | Cell membrane | Cytoplasm | 426 aa 161-164 |
| Q9ULK6 | RING finger protein 150 | Membrane | Potential signal peptide (1-34) | 438 aa 17-20 |
| P32239 | Gastrin/cholecystokinin type B receptor | Cell membrane | Membrane | 447 aa 179-192 |
| Q7Z4P5 | Growth/differentiation factor 7 | Extracellular | Potential signal peptide (1-19) | 450 aa 12-15 |
| Q9P109 | Beta-1,3-galactosyl-O-glycosyl-glycoprotein beta-1,6-N-acetylglucosaminyltransferase 4 | Golgi apparatus membrane | Lumen | 453 aa 24-27 |
| P21917 | D(4) dopamine receptor | Cell membrane | Membrane | 467 aa 160-163 |
| Q96GQ5 | UPF0420 protein C16orf58 | Membrane | Membrane | 468 aa 459-462 |
| Q96HE7 | ERO1-like protein alpha | Endoplasmic reticulum membrane  Peripheral membrane protein  Lumenal side | Signal peptide (1-23) | 468 aa 16-19 |
| P34969 | 5-hydroxytryptamine receptor 7 | Cell membrane | Membrane | 479 aa 207-210 |
| Q86YJ6 | Threonine synthase-like 2 | Extracellular | Extracellular | 484 aa 327-330 |
| Q400G9 | Archaemetzincin-1 | Uncertain | Uncertain | 498 aa 66-69 |
| Q9NQH7 | Probable Xaa-Pro aminopeptidase 3 | Mitochondrion | Mitochondrion | 507 aa 3-6 |
| Q8NC44 | Protein FAM134A | Membrane | Membrane | 543 aa 94-97 |
| Q8IWB1 | Inositol 1,4,5-trisphosphate receptor-interacting protein | Cell membrane | Uncertain | 547 aa 380-383 |
| O15245 | Solute carrier family 22 member 1 | Cell membrane | Cytoplasm | 554 aa 288-291 |
| Q8WXF7 | Atlastin-1 | [Endoplasmic reticulum membrane](http://www.uniprot.org/locations/SL-0097)  [Golgi apparatus membrane](http://www.uniprot.org/locations/SL-0134) | Cytoplasm | 558 aa 303-306 |
| Q86U10 | 60 kDa lysophospholipase | Uncertain | Uncertain | 573 aa 383-386 |
| Q86VW1 | Solute carrier family 22 member 16 | Cell membrane | Uncertain | 577 aa 294-297 |
| Q8WW52 | Protein FAM151A | Membrane | Uncertain | 585 aa 260-263 |
| Q8IYA8 | Coiled-coil domain-containing protein 36 | Uncertain | Uncertain | 594 aa 545-548 |
| Q9HA90 | Coiled-coil domain-containing protein 48 | Uncertain | Uncertain | 598 aa 29-32 |
| Q9UBU9 | Nuclear RNA export factor 1 | Cytoplasm  Nucleus | Cytoplasm  Nucleus | 619 aa 134-137 |
| Q5TGY1 | Transmembrane and coiled-coil domain-containing protein 4 | Membrane | Uncertain | 634 aa 464-467 |
| O95677 | Eyes absent homolog 4 | Cytoplasm  Nucleus | Cytoplasm  Nucleus | 639 aa 81-84 |
| P51168 | Amiloride-sensitive sodium channel subunit beta | Cell membrane | Membrane | 640 aa 517-520 |
| Q8N9H9 | Uncharacterized protein C1orf127 | Uncertain | Uncertain | 656 aa 39-42 |
| Q15067 | Peroxisomal acyl-coenzyme A oxidase 1 | Peroxisome | Peroxisome | 660 aa 123-126 |
| Q9Y2J8 | Protein-arginine deiminase type-2 | Cytoplasm | Cytoplasm | 665 aa 69-72 |
| O15296 | Arachidonate 15-lipoxygenase B | Cytoplasm | Cytoplasm | 676 aa 608-611 |
| Q8NE28 | Protein kinase-like protein SgK071 | Uncertain | Uncertain | 680 aa 529-532 |
| Q9UKF6 | Cleavage and polyadenylation specificity factor subunit 3 | Nucleus | Nucleus | 684 aa 105-108 |
| P54803 | Galactocerebrosidase | Lysosome | Signal peptide (1-42) | 685 aa 4-7 |
| Q8NC60 | Nitric oxide-associated protein 1 | [Mitochondrion inner membrane](http://www.uniprot.org/locations/SL-0168)  (m[atrix side](http://www.uniprot.org/locations/SL-9913)) | Uncertain | 698 aa 183-186 |
| Q6ZPD9 | Protein dpy-19 homolog 3 | Membrane | Membrane | 716 aa 179-182 |
| Q9UHD2 | Serine/threonine-protein kinase TBK1 | Cytoplasm | Cytoplasm | 729 aa 9-12 |
| P40879 | Chloride anion exchanger | Cell membrane | Uncertain | 764 aa 72-75 |
| Q76B58 | Protein FAM5C | Extracellular | Extracellular | 766 aa 46-49 |
| Q9NP78 | ATP-binding cassette sub-family B member 9 | Lyzosome  Membrane | Uncertain | 766 aa 135-138 |
| O43511 | Pendrin | Membrane | Cytoplasmic | 780 aa 83-86 |
| Q6PJG6 | BRCA1-associated ATM activator 1 | Nucleus | Nucleus | 821 aa 477-480 |
| Q13387 | C-Jun-amino-terminal kinase-interacting protein 2 | Cytoplasm | Cytoplasm | 824 aa 349-352 |
| A2RRH5 | WD repeat-containing protein 27 | Uncertain | Uncertain | 827 aa 569-572 |
| Q7RTY8 | Transmembrane protease serine 7 | Cell membrane | Extracellular | 843 aa 640-643 |
| B1AK53 | Espin | [Cell projection](http://www.uniprot.org/keywords/KW-0966) [Cytoplasm](http://www.uniprot.org/keywords/KW-0963) [Cytoskeleton](http://www.uniprot.org/keywords/KW-0206) | Uncertain | 854 aa 88-91 |
| Q5JQC9 | A-kinase anchor protein 4 | [Cell projection](http://www.uniprot.org/keywords/KW-0966) [Cilium](http://www.uniprot.org/keywords/KW-0969) [Flagellum](http://www.uniprot.org/keywords/KW-0282) | Potential propeptide (1-188) | 854 aa 113-116 |
| Q08043 | Alpha-actinin-3 | Cytoplasm  Actin filament  Focal adhesion  Pseudopodium | Uncertain | 901 aa 395-398 |
| P57737 | Coronin-7 | Cytoplasm | Cytoplasm | 925 aa 853-856 |
| Q6IWH7 | Anoctamin-7 | Cell membrane | Extracellular | 933 aa 403-406 |
| Q96DN2 | von Willebrand factor C and EGF domain-containing protein | Extracellular | Extracellular | 955 aa 335-338 |
| Q93033 | Immunoglobulin superfamily member 2 | Cell membrane | Extracellular | 1021 aa 783-786 |
| Q96RT8 | Gamma-tubulin complex component 5 | Cytoplasm | Cytoplasm | 1024 aa 276-279 |
| Q9HC29 | Nucleotide-binding oligomerization domain-containing protein 2 | Cytoplasm | Cytoplasm | 1040 aa 59-62 |
| Q7Z410 | Transmembrane protease serine 9 | Cell membrane | Extracellular | 1059 aa 538-541 and 862-865 |
| P42702 | Leukemia inhibitory factor receptor | Cell membrane | Potential signal peptide (1-44) | 1097 aa 28-31 |
| P53396 | ATP-citrate synthase | Cytoplasm | Cytoplasm | 1101 aa 49-52 |
| Q8TCU5 | Glutamate [NMDA] receptor subunit 3A | Cell membrane | Potential signal peptide (1-23) | 1115 aa 8-11 |
| Q96AE7 | Tetratricopeptide repeat protein 17 | Uncertain | Uncertain | 1141 aa 21-24 |
| *Staphylococcus aureus* subsp. aureus NCTC 8325, taxid: 93061 | | | | |
| **Q2FXB3** | Lantibiotic epidermin biosynthesis protein EpiB, putative | Uncertain | Uncertain | 997 aa 330-333 |
| **Q2FV72** | Ferrous iron transport protein B, putative | Uncertain | Uncertain | 664 aa 526-529 |
| **Q2G151** | Putative uncharacterized protein SAOUHSC_00304 | Uncertain | Uncertain | 333 aa 164-167 |
| **Q2FXI1** | Putative uncharacterized protein SAOUHSC_01866 | Uncertain | Uncertain | 241 aa 143-146 |

**5. References**

**1. Fehlhammer H, Bode W, Huber R (1977) Crystal structure of bovine trypsinogen at 1-8 A resolution. II. Crystallographic refinement, refined crystal structure and comparison with bovine trypsin. J Mol Biol 111: 415-438.**

**2. Prasad L, Leduc Y, Hayakawa K, Delbaere LT (2004) The structure of a universally employed enzyme: V8 protease from Staphylococcus aureus. Acta Crystallogr D Biol Crystallogr 60: 256-259.**

**3. Wang D, Bode W, Huber R (1985) Bovine chymotrypsinogen A X-ray crystal structure analysis and refinement of a new crystal form at 1.8 A resolution. J Mol Biol 185: 595-624.**

**4. Katz BA, Mackman R, Luong C, Radika K, Martelli A, et al. (2000) Structural basis for selectivity of a small molecule, S1-binding, submicromolar inhibitor of urokinase-type plasminogen activator. Chem Biol 7: 299-312.**

**5. Laxmikanthan G, Blaber SI, Bernett MJ, Scarisbrick IA, Juliano MA, et al. (2005) 1.70 A X-ray structure of human apo kallikrein 1: structural changes upon peptide inhibitor/substrate binding. Proteins 58: 802-814.**
